# Altitudinal Zonation of Green Algae Biodiversity in the French Alps

**DOI:** 10.3389/fpls.2021.679428

**Published:** 2021-06-07

**Authors:** Adeline Stewart, Delphine Rioux, Fréderic Boyer, Ludovic Gielly, François Pompanon, Amélie Saillard, Wilfried Thuiller, Jean-Gabriel Valay, Eric Maréchal, Eric Coissac

**Affiliations:** ^1^Laboratoire de Physiologie Cellulaire et Végétale, CEA, CNRS, INRAE, IRIG, Université Grenoble Alpes, Grenoble, France; ^2^Jardin du Lautaret, CNRS, Université Grenoble Alpes, Grenoble, France; ^3^Université Grenoble Alpes, Université Savoie Mont Blanc, CNRS, LECA, Grenoble, France

**Keywords:** *Chlorophyta*, metabarcoding, mountain environment, soil, biodiversity, high elevation, *Sanguina*, snow algae

## Abstract

Mountain environments are marked by an altitudinal zonation of habitat types. They are home to a multitude of terrestrial green algae, who have to cope with abiotic conditions specific to high elevation, e.g., high UV irradiance, alternating desiccation, rain and snow precipitations, extreme diurnal variations in temperature and chronic scarceness of nutrients. Even though photosynthetic green algae are primary producers colonizing open areas and potential markers of climate change, their overall biodiversity in the Alps has been poorly studied so far, in particular in soil, where algae have been shown to be key components of microbial communities. Here, we investigated whether the spatial distribution of green algae followed the altitudinal zonation of the Alps, based on the assumption that algae settle in their preferred habitats under the pressure of parameters correlated with elevation. We did so by focusing on selected representative elevational gradients at distant locations in the French Alps, where soil samples were collected at different depths. Soil was considered as either a potential natural habitat or temporary reservoir of algae. We showed that algal DNA represented a relatively low proportion of the overall eukaryotic diversity as measured by a universal Eukaryote marker. We designed two novel green algae metabarcoding markers to amplify the *Chlorophyta* phylum and its *Chlorophyceae* class, respectively. Using our newly developed markers, we showed that elevation was a strong correlate of species and genus level distribution. Altitudinal zonation was thus determined for about fifty species, with proposed accessions in reference databases. In particular, *Planophila laetevirens* and *Bracteococcus ruber* related species as well as the snow alga *Sanguina* genus were only found in soil starting at 2,000 m above sea level. Analysis of environmental and bioclimatic factors highlighted the importance of pH and nitrogen/carbon ratios in the vertical distribution in soil. Capacity to grow heterotrophically may determine the *Trebouxiophyceae* over *Chlorophyceae* ratio. The intensity of freezing events (freezing degree days), proved also determinant in *Chlorophyceae* distribution. Guidelines are discussed for future, more robust and precise analyses of environmental algal DNA in mountain ecosystems and address green algae species distribution and dynamics in response to environmental changes.

## Introduction

Green algae are unicellular, colonial or multicellular photosynthetic organisms that are ubiquitous in almost all ecosystems. They have evolved in two major lineages, one referred to as *Chlorophyta*, what has been traditionally called green algae, another referred to as *Charophyta*, containing a smaller but often geographically widespread number of taxa (Lewis and Mccourt, 2004; Domozych et al., 2016). Recently, a third lineage was introduced, covering marine *Prasinodermophyta*, which diverged before the split of *Chlorophyta* and *Streptophyta* (Li et al., 2020), and that was not considered in the present work.

The majority of green algae species are found in aero-terrestrial habitats, living either freely or in lichens in association with fungi (Rindi et al., 2010), from wet to dry areas (Lewis and Mccourt, 2004; Holzinger et al., 2017). Although algae are often considered to live primarily in “free” water, soil surface is known for long to host an important algal biodiversity (Tchan, 1952; Reisigl, 1969; Koelewijn et al., 2001; John et al., 2011; Foets et al., 2020). Numerous green algae can grow heterotrophically in the dark, using an external source of organic carbon, and often much faster compared to pure autotrophic conditions (Fan et al., 2012; Bell, 2013). Soil can therefore be colonized from its surface to its depth by the combined photosynthetic and heterotrophic capacity of algal communities. In soil, algae contribute actively to the global cycles of carbon and nitrogen (Elbert et al., 2012). In addition, soil may also contain resting algal cysts or non-motile spores (aplanospores), thus acting as a reservoir for species needing more appropriate conditions to grow. This latter context is difficult to assess, since most microbial species are non-cultivable (Schmeisser et al., 2007) and the determination of life cycles are therefore extremely challenging. Some algae species can also occupy non-liquid water systems, most notably snow and ice (Remias, 2012; Lukes et al., 2014; Hisakawa et al., 2015; Holzinger et al., 2016; Lu et al., 2016; Hoham and Remias, 2019).

Mountain environments are marked by the tight apposition of contrasted habitats, structured by the topography, elevation, temperature, exposure to sunlight, wind, precipitations, etc., with abiotic conditions considered as “extreme” such as high light intensity, extreme negative temperatures, high UV irradiance, strong winds, desiccation, extreme diurnal variations in temperatures and chronic scarceness of nutrients (Geremia et al., 2016). Knowledge on the biodiversity and distribution of green algae in mountain environments is fragmented and overall poor. Some habitats have attracted more attention, such as lakes (Jacquemin et al., 2019) or snowpack at high elevation (Hoham and Remias, 2019). Soil was barely explored, although it represents a much more extended and permanent surface, with a strong potential for spatiotemporal studies of species distribution, interfaced with hydrologic networks and seasonal snow covers. A first survey of aero-terrestrial algae in alpine soils was based on the morphological study of cells sampled in the Tyrolean Alps above 3,000 m a.s.l., with about 90 species described, counting a majority of *Chlorophyta* (Reisigl, 1964). A recent review on algae populating soil surface in the Alps (Karsten and Holzinger, 2014), reported that eukaryotic algae of mountains were mainly represented by monadoid and coccoid *Chlorophyta*, and to a lower extent *Charophyta* and a few *Ochrophyta* (Gartner, 2004; Tschaikner et al., 2007). This review highlighted the potential role of UV light and desiccation as drivers of soil algal communities in the Alps (Karsten and Holzinger, 2014), but other environmental factors may be determinant as well.

In environments undergoing rapid evolutions, photosynthetic algae can act as pioneering organisms, able to develop autotrophically on empty surfaces, and being the primary producers allowing the subsequent foundation of trophic networks. As an example, green algae have been shown to be primary colonizers after glacier’s retreat (Kastovska et al., 2005; Hotaling et al., 2017). Atmospheric CO_2_ proved to influence microbial communities in a pH-dependent manner (Gulliver et al., 2016; Yu and Chen, 2019). It is also expected, although not demonstrated yet, that the current increase in atmospheric CO_2_ could be beneficial to the development of photosynthetic algae and as such, act positively on the efficiency of colonization. Green algae are therefore expected to be “markers” of climate change. In mature ecosystems, once established in their habitat, green algae can proliferate to such an extent that they form so called blooms, which can be determinant in further evolutions of their environment. For instance, at the surface of snowpack, green algae blooms are detected by the pigmentation of resistant cells (cysts), containing red carotenoids, such as astaxanthin (Holzinger et al., 2016). The “red snow” thus formed reduces the albedo, triggering an increase of superficial temperature and accelerating snow melting (Lutz et al., 2016; Di Mauro et al., 2020). In this aspect, green algae are therefore also expected to be “actors” of environmental changes. If we aim to comprehend the dynamics of ecosystems, data on microbial communities, and most notably on photosynthetic microorganisms, are critical. Spatiotemporal distribution of green algae is thus a major information we need to address changes in mountain areas exposed to drastic seasonal variations and to irreversible transformations triggered by climate evolution. These data are currently missing.

The distribution of plant species in mountain environment is generally assumed to be mostly constrained by abiotic factors (Benton, 2009; Martinez-Almoyna et al., 2019; Körner, 2021) and it could be the same for microalgae, as suggested by a microscopy-based study performed in the Himalayas along an elevational gradient (Rehakova et al., 2011). To study microalgae communities, some research groups have performed sampling, followed by microscopic observation of microalgae (e.g., Rehakova et al., 2011) or DNA-barcoding analyses (e.g., Hall et al., 2010). None of these studies has provided a comprehensive taxonomic assessment of sampled green algae. Cell morphology is not sufficient to identify species, and information on life cycles, biochemical traits or genomic sequences would be requested to refine characterizations. In addition, microscopy-based studies rely on the relative abundance of species at the time of sampling, and on the survival rate of species, when transferred from their environment to the laboratory. By contrast, environmental DNA allows detecting genomic fragments released by broken cells, by past and present communities, thus providing data on the spatial distribution with a so called “soil memory effect,” mitigating short term temporal variations (Foucher et al., 2020). DNA-based analysis is therefore currently the best compromise to obtain more exhaustive information on microalgae communities (Lutz et al., 2016; Groendahl et al., 2017; Foucher et al., 2020).

Metabarcoding is based on assigning amplified molecular operational taxonomic unit (MOTU) sequences from an environmental sample (eDNA) to a database of sequences. Its success depends on the effectiveness of the markers to amplify targeted taxa, the effectiveness of the PCRs and sequencing, the status of the reference database (Ficetola et al., 2010, 2016) and the quality of the bioinformatic pipeline (Calderòn-Sanou et al., 2020). Markers for green algae of the *Chlorophyta* phylum were designed in several studies (e.g., Vieira and Bagatini, 2016; Zou et al., 2016; Pfendler et al., 2018) but most previous works use more general eukaryotic markers like ITS (internal transcribed spacer, Heeger et al., 2018) or COX1 (cytochrome oxidase I, Ward et al., 2005). The use of general eukaryotic markers reflects the fact that databases contain more of these sequences available for assignments. None of these markers has proven ideal for the study of green algae. They have issues such as too high or too low variability, presence of introns, absence of data in the databases, or amplification of only a part of the community (Vieira and Bagatini, 2016).

**FIGURE 1:**
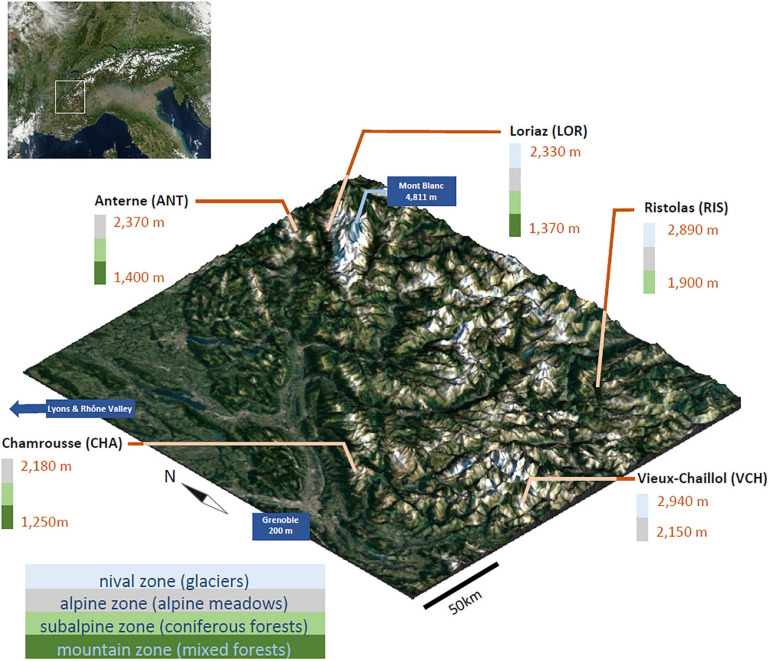
Aerial view of the five sampled elevational gradients and their topography (1,250–2,940 m). Selected sites cover the full range of environments and habitats found in the Alpine altitudinal zonation and a representative geographical distribution. DNA was extracted from soil collected at each sampling location and used for an evaluation of *Chlorophyta* communities by DNA metabarcoding. Satellite images from Nasa.

Here, we evaluated green algae biodiversity, focusing on *Chlorophyta*, in selected elevational gradients in the French Alps from 1,250 to 3,000 m high, from forests at lowest levels, to a variety of other habitats such as heathlands, grasslands and rocky areas at high elevations (Figure 1). Presence of green algae was monitored at different depths in the soil. The sampling campaign was part of a large project aiming at understanding biodiversity and its drivers and dynamics over time in the Alps, called Orchamp, and which provided the samples^1^. To that purpose, we validated two new markers for metabarcoding studies, a *Chlorophyta* phylum marker “Chlo01” designed in the V7 region of the 18S ribosomal RNA, to cover most of the green algae and a *Chlorophyceae* marker “Chlo02” designed in the 23S ribosomal RNA chloroplast sequence. We then asked whether green algae could be detected with these markers and if we could point some possible environmental drivers of their distribution patterns and community structure.

## Materials and Methods

### Soil Sampling

In late summer 2016, 158 soil samples were collected in the French Alps along elevation gradients covering elevations from 1,250 to 2,940 m at five different sites: Chamrousse (CHA) 45.098692°N, 5.885508°E, elevations from 1,250 to 2,180 m, sampled on September the 1st; Loriaz (LOR) 46.038079°N, 6.918759°E, from 1,370 to 2,330 m, sampled on September the 6th; Anterne (ANT) 46.009245°N, 6.805825°E from 1,400 to 2,370 m, sampled on September the 7th; Ristolas (RIS) 44.724622°N, 7.031392°E, from 1,900 to 2,890 m, sampled on September the 15th; and Vieux Chaillol (VCH) 44.721000°N, 6.187555°E, from 2,150 to 2,940 m, sampled on September the 14th (Figure 1). The four lowest sites include environments composed of forests at the lowest elevations, and grasslands and pastures at the highest elevations. At their highest elevations, only RIS and VCH reach the nival zone, and the latter has no forest at its base. The sampling was done along the elevational gradients approximatively every 200 m. For each level, sampling was performed in triplicate at two different soil horizons: the litter (soil not totally decomposed) between 0 and 10 cm depth, and the deep soil between 10 and 25 cm depth (soil totally decomposed).

### Environmental Variables

In addition to the sampling site (*Site*), six environmental variables were measured for each sample: elevation above sea level in meters (*Elevation*); soil pH (*pH*) and organic matter content (*Organic Matter*) assessed using standard protocols (Robertson et al., 1999); total soil carbon (*Carbon*) and nitrogen (*Nitrogen*) measured using a Flash EA1112 (Thermo Scientific) elemental analyzer (Martinez-Almoyna et al., 2019); the *Carbon* over *Nitrogen* ratio (*C/N ratio*). Ten bioclimatic variables, estimated as averages over 1988–2018 were used: the mean of the annual temperature (TG), the growing degree days (GDD), the freezing degree days (FDD), these three variables were estimated at 1 and 10 cm below ground, the climatic water stress (CWD), the solar radiation, the total snow depth (DSN_T_ISBA) and the diurnal temperature range (DRT.air). Details on these variables are available in Martinez-Almoyna et al. (2019) and Martinez-Almoyna et al. (2020) Finally, two categorical variables: the type of environment (*Environment*) with two modalities, forest or open-area, and the soil horizon (*Horizon*) with two modalities, litter or deep soil, complete this description. It is common for environmental variables to be multi-colinear with respect to each other. To overcome this problem, a subset of continuous environmental variables was selected using the variance and inflation factor (VIF) criterion: VIF = 1/(1–R^2^) where R^2^ is the coefficient of determination of the multiple linear model of one of the explanatory variables explained by the others. Variables with a VIF greater than 5 (Sheather, 2009) were iteratively removed. At the end of the selection process, *Elevation*, *pH*, *Nitrogen*, *C/N ratio*, *FDD* at 1 cm, *CWD*, and *DTR* were retained, while *other variables* were removed because of there colinearity with the selected variables (Supplementary Figure 1).

### DNA Metabarcoding Markers

Two new DNA metabarcodes were designed for this analysis. The first one targets *Chlorophyta* (Chlo01), the second targets Chlorophyceae (Chlo02). To design these new metabarcodes, 1,628 complete chloroplast sequences were downloaded from the NCBI database^2^ (November 2017) comprising 74 Chlorophyta plastid genomes, including 23 Chlorophyceae genomes. The ecoPCR software^3^ and the ROBIBarcodes R package^4^ were used to refine the corresponding primer sequences, assess the conservation of the priming sites using sequence logos (Schneider and Stephens, 1990), and estimate *in silico* the taxonomic resolution as proposed in Ficetola et al., 2010. The sequence library used to realize those tests was the entire EMBL sequence database (release 139, Amid et al., 2020). The hybridization temperature was empirically determined using OligoCalc^5^ following recommendations in Taberlet et al. (2018). Chlo01 was adapted from the eukaryotic marker Euka02 and corresponds to the V7 variable region of nuclear 18S rRNA gene (Taberlet et al., 2018). Corresponding primers were modified to make them specific of Chlorophyta. Chlo02 was designed using the ecoPrimers software^6^ (Riaz et al., 2011). A third marker, Euka03 (Euka03F: CCCTTTGTACACACCGCC, Euka03R: CTTCYGCAGGTTCACCTAC) targeting all eukaryota, was used to assess relative proportion of algal eDNA within the eukaryota super kingdom (Taberlet et al., 2018).

### Algae Mock Community for Positive Controls

A mock community constituted by 13 green unicellular marine algae species from the Roscoff Culture Collection (RCC)^7^ were used as template for the PCR positive controls. According to the order presented in Supplementary Table 1, the DNA concentration for each species was adjusted to half of the previous one. The community was expected to be similar in concentration and complexity to our samples. If the thirteen species could theoretically be amplified by Euka03 and Chlo01, only 3 species were expected to be amplified by Chlo02. The DNA of each species constituting the mock community was extracted using the Macherey Nagel NucleoSpin Plant II extraction kit according to the instruction manual^8^.

### Soil DNA Extraction, PCR Amplification and Sequencing

Extracellular DNA was extracted from 15 g of soil or litter as described previously (Taberlet et al., 2012). PCRs were then performed in triplicates for each sample, in parallel with extraction blanks, with no template soil and PCR blanks, with no template DNA (negative controls) and positive control. After an initial denaturation at 95°C for 10 min, 40 cycles (38 for Chlo01) of amplification were run: denaturation 95°C, 30 s; hybridization 30 s; elongation 72°C 1 min. Hybridization temperature was respectively 55°C, 50°C, and 55°C for Chlo01, Chlo02, and Euka03. Each PCR product was individually tagged according to Taberlet et al. (2018). This enabled the pooling of up to 1,052 PCRs per sequencing libraries. Pooled PCRs were purified using the Qiagen MinElute PCR Purification Kit for Chlo01 and Euka03 and the Qiagen QIAquick PCR Purification Kit for Chlo02^9^. Sequencing libraries were prepared and sequenced (2 × 125 bp paired-end reads) by Fasteris (Geneva, Switzerland), using their MetaFast protocol.

### Design of a Reference Database of Green Algae Sequences

The reference sequence databases used for taxonomic assignment were extracted using ecoPCR (Ficetola et al., 2010; Boyer et al., 2016) from the EMBL database (version 140, 2019; Amid et al., 2020), using Chlo01, Chlo02 or Euka03 primers as queries. EcoPCR results were filtered using OBITools (Boyer et al., 2016) to keep only the sequences annotated with an unambiguous family and genus. Strictly identical sequences were merged and their taxonomic annotations summarized at the lowest common ancestor. Sequences containing ambiguous nucleotides were also discarded. The cleaned reference databases for Chlo01, Chlo02, and Euka03 are constituted respectively by 1444, 744, and 17207 sequences. The Chlo01 database represents 295 genera and 62 families belonging the *Chlorophyta*. The Chlo02 database represents 42 genera and 19 families belonging the *Chlorophyceae*. The Euka03 database represents 5179 genera and 2488 families belonging the *Eukaryota*.

### Read Filtering and Processing

The reading pairs were assembled, and demultiplexed to be separated by sample. The sequences were then de-replicated to obtain the number of reads of each sequence variant in each PCR. These steps and the following were realized using the OBITools software (Boyer et al., 2016) following the protocol by Taberlet et al. (2018). According to the amplicon lengths estimated from our reference databases for each marker, sequences shorter than 65 bp and longer than 200 bp for Chlo01 and Euka03, and 130 bp for Chlo02 were discarded. Rare sequence variants never represented by more than 10 reads in a PCR were discarded. Punctual errors generated during PCR cycles were discarded using the obiclean (Boyer et al., 2016). Sequence variants were taxonomically annotated using the ecotag and the reference database described above. Only MOTUs annotated in the target clade of its marker were conserved. At this stage, any MOTU that was more abundant in the negative PCR controls than in any of the samples was annotated as a contaminant and discarded.

### Removing of Unsuccessful PCRs

Of all the PCRs analyzed, some provided unreliable results. They were detected according to two criteria, the number of reads associated with a PCR, and considering a sample, the similarity between PCR replicates. Based on the distribution of the number of reads per PCR observed for each marker, PCRs with more than 200 reads for the markers Chlo01 and Chlo02, and 1,000 reads for Euka03 were considered unsuccessful and rejected. The reproducibility of PCR replicates was estimated by the distance between a replicate and the barycenter of the replicates for that sample. Distances were estimated using Euclidean distances computed on the Hellinger transformed data (square roots of the relative frequencies), which corresponds to a correlation distance. Distribution of these distances is used to detect potential outliers.

### Data Analyses and Statistics

Further filtering and data analysis were run using R (v.3.6.2, R Development Core Team, 2019) using the ROBItools package^10^ for managing OBITools data files, ggplot2 (Wickham, 2011) for graphics, the ade4 package (Dray and Dufour, 2007) for every multidimensional scaling and the Vegan package (Oksanen et al., 2020) for computing Hellinger transformation (square rooted relative frequencies), relative frequencies, and *Permutational multivariate analyses of variance* (PERMANOVA). The iteratively reweighted least squares (IRLS) procedure for estimating outlier robust linear models was computed with the robustRegBS function of the robustreg package, implementing the methods presented in Hubert (1981).

### Taxonomic Diversity

The diversity of algal communities was estimated for metabarcoding data using Hill numbers, with *q* = 1 here (the exponential of the Shannon entropy index). A Hill number is the effective number of species composing a theoretical community, which would be perfectly even, and having the same diversity as the community studied. A taxonomic diversity measured by a Hill number (^*q*^D) takes less and less account of rare species when the q parameter increases. In the case of metabarcoding data, using *q* = 1 penalizes not only the rarest species, but also the many false taxa generated during PCR amplification that occur at low read frequencies. As a result, the taxonomic diversity^1^*D* values estimated from DNA metabarcoding data are relatively congruent with those estimated from conventional inventories (Calderòn-Sanou et al., 2020). The relationships between diversity and environmental parameters were measured by discretizing the gradients into seven levels. The strength of the relationship was estimated with a one-factor ANOVA and its significance was tested with the Kruskal–Wallis method.

### Community Turnover

The composition turnover between communities was estimated using Euclidean distances calculated on the Hellinger transformed contingency table of sequence reads, per MOTU and samples. We then projected those pairwise distances using principal coordinate analysis (PCOA). The strength of the correlations between community changes and environmental variables was estimated using Redundancy Analysis (RDA) using the Vegan R package. The environmental variables were centered and scaled for the analysis. The optimal model was selected using a forward-backward selection procedure implemented in the ordistep function. Partitioning of the community changes variance was performed using the varpart function. Permutation-based estimate of *p*-values relied on 999 permutations.

### Niche Inference

Niches of the MOTUs identified at the species or genus levels were estimated using the Outlying Mean Index (OMI) method (Doledec et al., 2000) as implemented in the niche function of the ADE4 R package. This method describes the niche according to three terms: its marginality, its marginal tolerance and its residual tolerance. Marginality measures the distance of the center of a taxon’s niche from the center of the environmental space, which would represent a ubiquitous species. Marginal tolerance measures the width of the niche along its the marginality axis, defined by the vector connecting the center of the environmental space and the center of the taxon’s niche. The marginal tolerance measures the width of the niche in the orthogonal plan to the marginality axis. Doledec et al. (2000) measured the specialization of a taxon by the non-zero marginality of its niche. In our case a taxon could also be considered as specialized, if its marginality was null but its marginal tolerance was lower than that expected for a taxon uniformly distributed in the environmental space. Therefore, a taxon was defined as specialized if its marginality was not null or if its marginal tolerance is smaller than expected under uniform distributions. Both conditions were tested by permutation (*n* = 999) following the procedure implemented in the *r*-test function.

## Results

### Design and Validation of *Chlorophyta* and *Chlorophyceae* DNA Markers

There are three main lineages of green algae: *Chlorophyta*, *Prasinophyta*, and *Charophyta*, the latter of which also is close to land plants (Kapraun, 2007). We focused on the *Chlorophyta* phylum, an important and diverse lineage of green algae, and were particularly interested in the *Chlorophyceae* class, which we expected to yield the greatest diversity of algae. The developed markers were termed Chlo01 and Chlo02.

Similarly to Euka02 (Taberlet et al., 2018), the Chlo01 marker corresponds to the V7 region of the 18S nuclear rRNA gene. Its length ranges from 80 to 180 bp. The primer pair Chlo01F: AGTTGGTGGGTTGCCTTGT, Chlo01R: CACAGACCTGTTATTGCCTC has an estimated hybridization temperature of 55°C. The Chlo01 marker theoretically discriminates 24% of the sequences at the species level, 43% at the genus level, 53% at the family level, 62% at the order level and 74% at the class level (Supplementary Figure 2).

The Chlo02 marker corresponds to a sequence included in the 23S chloroplastic rRNA gene. Its length ranges from 91 to 94 bp. The primer pair Chlo02F: RCTTAGTCCCGGCCATT, Chlo02R: CTAAGTGGWAAAGGATGTG has an estimated hybridization temperature of 50°C. The Chlo02 marker discriminates 47% of the sequences at the species level, 65% at the genus level, and 73% at the family level (Supplementary Figure 2).

### Sequencing Results

We used the three markers to amplify DNA extracted from soil samples collected along the five elevation gradients. After filtering, the Chlo01 marker amplified 4,080 MOTUs represented by ∼5.8 million reads, including 566 Chlorophyta MOTUs corresponding to 3.3 million reads (see Table 1). The Chlo02 marker amplified 8,580 MOTUs, corresponding to 6.3 million reads; among them, 61 MOTUs belonged to Chlorophyceae, represented by less than 0.2 million reads. The Euka03 marker amplified 8,743 MOTUs, represented by 5.7 million reads, including 4,108 *Eukaryota* MOTUs corresponding to 4.1 million reads and 37 *Chlorophyta* MOTUs representing only 14,829 reads.

**TABLE 1:** Amplified MOTU and read counts by each marker for all samples.

Marker	Number of MOTU and reads in raw data	Number of MOTU and reads after filtering	Number of green algae MOTU and reads
Chlo01	1,280,988 MOTUs 12,849,650 reads	4,080 MOTUs 5,763,181 reads	566 MOTUs 3,305,241 reads
Chlo02	2,392,669 MOTUs 32,640,026 reads	8,580 MOTUs 6,210,043 reads	61 MOTUs 186,616 reads
Euka03	1,902,234 MOTUs 13,604,341 reads	8,743 MOTUs 5,700,798 reads	37 MOTUs 14,829 reads

### *Chlorophyta* DNA Represents a Minor Fraction of Soil DNA

**FIGURE 2:**
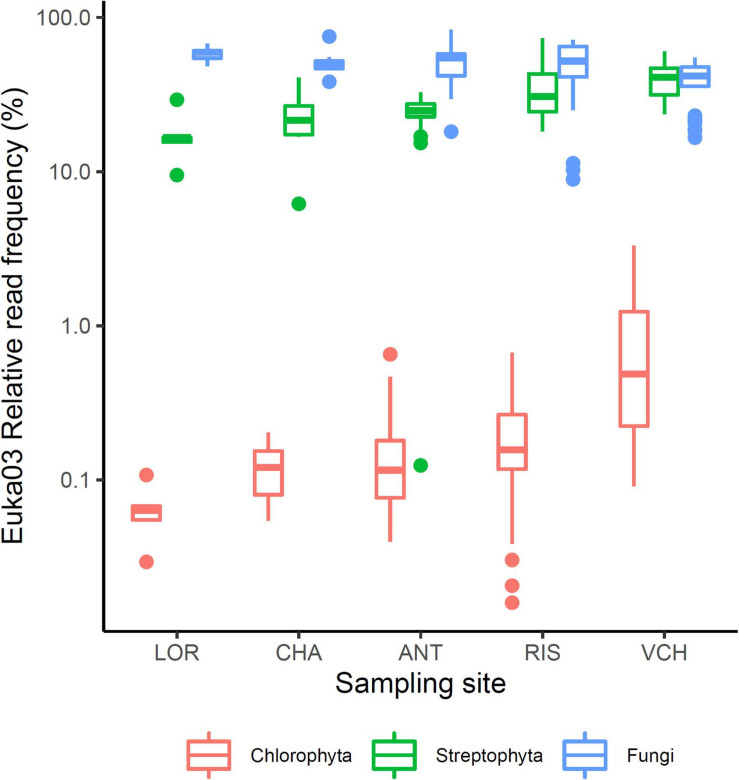
Relative frequencies of three eukaryotic clades; *Fungi*, *Streptophyta*, and *Chlorophyta*, among the sampling sites. The lower and upper limits of the boxes correspond to the first and third quartile, respectively, while the bold center line marks the median. The whiskers delineate the confidence interval defined as 1.5 times the difference between the first and third quartile. Outlier PCRs ranging outside of that interval are marked with dots.

**FIGURE 3:**
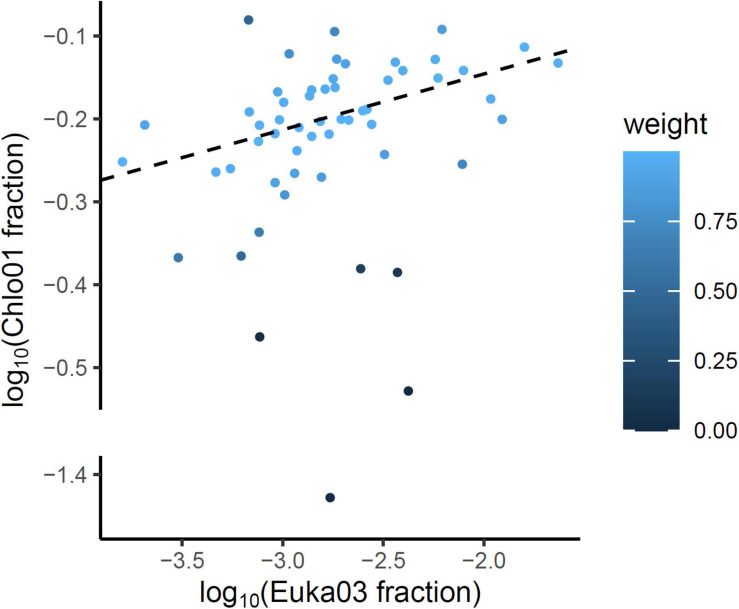
On a logarithmic scale, the relative frequencies of *Chlorophyta* reads estimated by the two markers Chlo01 and Euka03 are correlated. The linear model (dotted line) was estimated using an iteratively reweighted least squares procedure (IRLS) to underweight the influence of the few outliers PCRs. The blue scale indicates the weight associated to each PCR after the convergence of the algorithm.

To evaluate the relative part of algal eDNA present in soil samples, data obtained with the Euka03 marker were analyzed. Figure 2 shows the fraction of fungi, *Streptophyta* (mostly vascular plants) or *Chlorophyta* reads amplified by the Euka03 marker. While fungi and vascular plants occupy on average a high fraction of the reads, 59% (sd = 19%) and 21% (sd = 17%), respectively, *Chlorophyta* represent less than 3.3% of the reads in every PCR and 0.6% on average. In fact, only 18.6% of the PCRs with the Euka03 marker had some *Chlorophyta* reads (Figure 2). That trend is the same at every sampling site, even if the abundance of algae seems to increase at sites that cover higher elevations. Due to this low abundance of reads, which could result in under-sampling of diversity, and due to the low taxonomic resolution of Euka03, only 37 MOTU of *Chlorophyta* were identified. Of these, 7 belong to *Chlorophyceae* and 16 to *Trebouxiophyceae*. That low abundance of *Chlorophyta* eDNA is also confirmed by the marker Chlo01. Despite the fact that this marker is supposed to be highly specific to that clade (Supplementary Figure 2), we observed that many sequences were not annotated as *Chlorophyta*. Such high artifactual amplifications are commonly observed when target DNA concentration is very low in PCRs. Using an IRLS procedure, a linear model explaining 25% of the variance can be established on a logarithmic scale between the relative frequencies of *Chlorophyta* reads estimated by Euka03 and Chlo01 (Figure 3).

### Diversity of the *Chlorophyta* Communities

**FIGURE 4:**
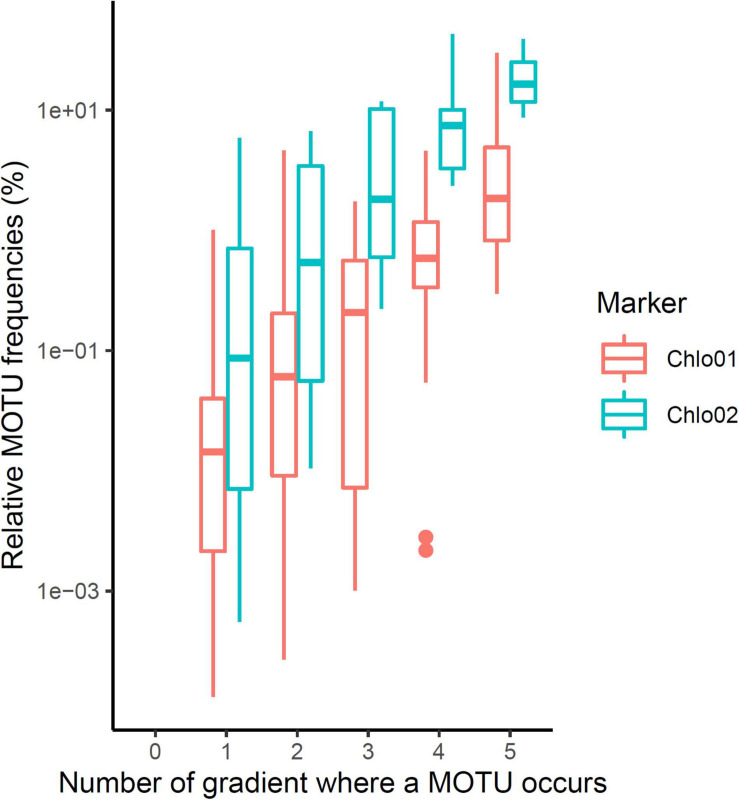
Endemism of the MOTUs according to their maximum frequency of occurrence in one of the gradients. The higher a MOTU has a high frequency of reads on at least one gradient, the more likely it will be present in many gradients.

**FIGURE 5:**
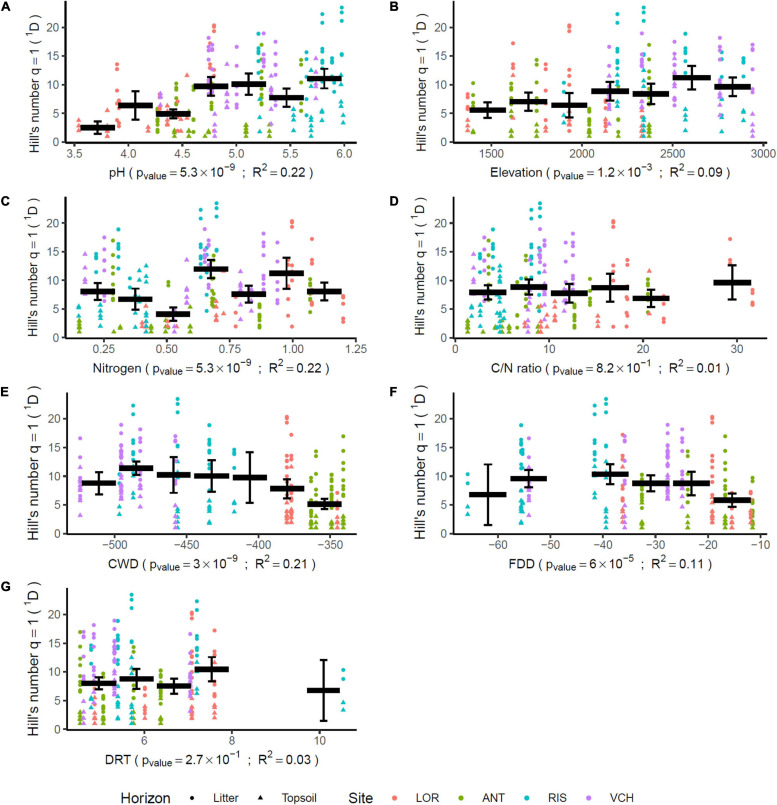
Impact of environmental parameters on algae community diversity. Each gradient is divided in seven parts. This division allows for mixing in a single part, samples from several sites. The bold horizontal bars indicate the mean of the diversity for the covered interval. The whiskers delimitate the 95% confidence interval of that mean. The *p*-values are adjusted for multiple tests using the false discovery rate method (Benjamini and Hochberg, 1995). The determination coefficients *R*^2^ measure the part of the variance explained by the sliced gradients using one way ANOVA.

The scarcity of *Chlorophyta* eDNA, confirmed by both Euka03 and Chlo01, can be explained either by a low biomass of algae in Alpine terrestrial environments or by our poor ability to extract algal eDNA from soil samples. Whatever the reason, this rarity limits the completeness of our sampling. Therefore, we certainly sampled only the most abundant taxa. This must be kept in mind when analyzing the data. For checking at minima, the quality of the^1^D estimation for algae and considering our data filtering stringency, we estimated^1^D for positive controls carried out on the mock community of 10 marine species of *Chlorophyta*. According to its composition, the theoretical diversity of the mock community was^1^*D* = 4.0 using the marker Chlo01 and 1.8 using the marker Chlo02 since only three of the ten species are *Chlorophyceae*. For Chlo01 the 72 replicates of the positive control gave a mean diversity^1^*D* = 3.83 species (sd = 0.016), which is slightly underestimated. For Chlo02, the same positive controls gave a mean diversity^1^*D* = 1.330 species (sd = 0.0016) instead of the theoretical 1.8. Over all five elevation gradients, the mean diversity observed for a sample for *Chlorophyta* (Chlo01) was^1^*D* = 9.58 (sd = 0.018 for 321 PCRs), and for *Chlorophyceae* (Chlo02)^1^*D* = 2.224 (sd = 0.0075 for 187 PCRs). A rough estimate of regional diversity (ɤ), by cumulating the results of the five elevational gradients, gave^1^*D* = 49.03 for *Chlorophyta* and^1^*D* = 13.40 for *Chlorophyceae*. The β diversity estimated as ɤ/α can be evaluated to 5.11 sites for Chlo01 and 6.02 sites for Chlo02. These two values have to be related to the number of studied gradients, i.e., five, and indicate that most of the MOTUs are site specific. Among the 566 MOTUs identified by Chlo01, 367 are present on only one gradient, 76 on two, the remaining 123 being observable on at least three gradients. For Chlo02, among the 61 MOTUs detected, 35, 9 and 17 MOTUs appear respectively in one, two, or three or more gradients (Figure 4). There is a strong link in that dataset between the endemism of a MOTU and its rarity, the MOTUs occurring in one or two sites only are also those having the lowest frequencies of occurrences at these sites (Figure 4). Therefore, the high endemism observed was probably related to the low coverage of the sampling. With the exception of the CHA gradient, which shows atypical results, among the seven non-collinear environmental and bioclimatic variables, pH, elevation, nitrogen, CWD and FDD (Figure 5) are significantly related to diversity. They explained 22, 9, 22, 21, and 11% of the variance of^1^D, respectively.

At the 5% threshold, the C/N ratio and DRT have no detectable effect on algal community diversity. The litter, which was richer in nitrogen, carbon and organic matter had a mean algal diversity^1^*D* = 11.8 (sd = 0.43) significantly higher (Mann–Whitney *p*-value = 10^–15^) than that of the deep soil layer^1^*D* = 6.7 (sd = 0.43). On the other hand, forest environments did not present a significantly different diversity from open environments (Mann–Whitney *p*-value = 0.54), although the former were also richer than the latter in Nitrogen, Carbon and Organic Matter.

### Main Components of *Chlorophyta* Communities

**FIGURE 6:**
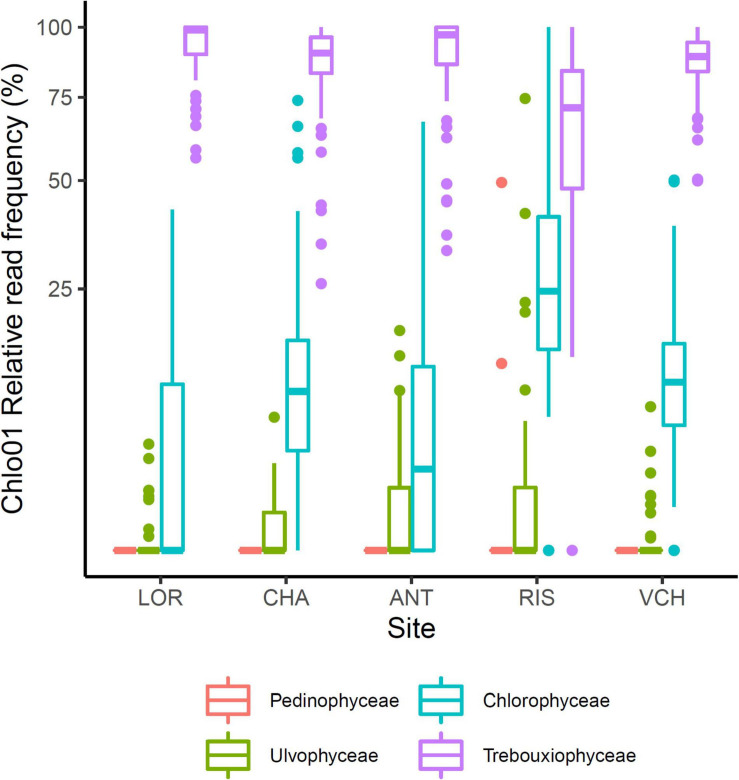
Distribution of the four taxonomic classes of *Chlorophyta*. *Trebouxiophyceae* and *Chlorophyceae* are the two main clades. *Pedinophyceae* just occurs sporadically in two PCRs on the RIS gradient.

**FIGURE 7:**
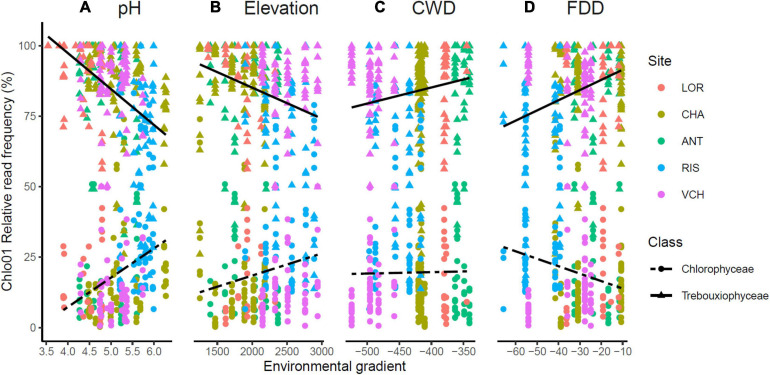
*Trebouxiophyceae* and *Chlorophyceae* relative abundance was impacted by soil pH **(A)**, elevation **(B)**, CWD **(C)**, and FDD **(D)**. The upper lines indicate the tendency of *Trebouxiophyceae* relative abundance when the environmental (pH, elevation) or bioclimatic (CWD, FDD) values increase. The opposite trend is materialized for the *Chlorophyceae* by the bottom dashed lines.

The *Chlorophyta* taxa identified with Chlo01 belong to four classes: *Trebouxiophyceae*, *Chlorophyceae*, *Ulvophyceae*, and *Pedinophyceae*. They corresponded respectively to 82.3, 11.1, 1.6, and 0.02% of the reads of this marker. *Pedinophyceae*, the rarest clade, was detected only on the RIS gradient in only two PCRs (Figure 6). *Trebouxiophyceae*, and *Chlorophyceae*, the two most abundant classes see their relative abundance evolving as a function of soil pH, elevation, CWD, and FDD (Figure 7). Because of their large dominance and the relative measure of their abundance, the decrease of one class mechanically increased the other. It was therefore not possible from these results to decide between the different hypotheses of substitution of one class by the other, the rarefaction of one class, or the increase of the other. As for the variation in diversity presented below, environmental factors had a significant effect, with here, again, a higher variance explained by pH (*R*^2^ = 0.22) than that explained by elevation (*R*^2^ = 0.026). On the other hand, no effect of nitrogen or C/N ratio was detected on this variation in abundance between the two classes.

### The Impact of Environmental and Bioclimatic Variables Was Significant but Small

**FIGURE 8:**
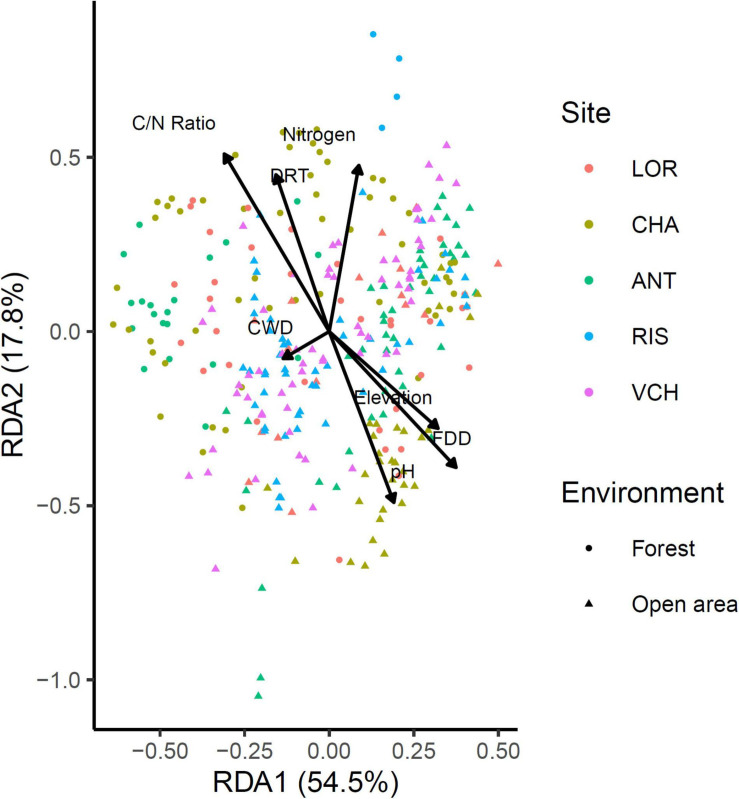
Redundancy analysis (RDA) of the *Chlorophyta* community against seven environmental (Nitrogen, Elevation, C/N ratio, pH) and bioclimatic (DRT, CWD, FDD) variables.

The impact of environmental variables on *Chlorophyta* community variation was assessed using a RDA on non-scaled Hellinger transformed community data (Figure 8). Sites were used as a covariate. Algae species may use various sources of organic carbon available in soil (Pintaldi et al., 2021), as part of their heterotrophic and/or mixotrophic life styles, and nitrogen, including soluble nitrate, nitrite and/or ammonium, as well as colloid-bound ammonium (Lee et al., 2006). In addition, the bioavailability of inorganic nitrogen is influenced by soil pH (Lee et al., 2006). The model selection retained the seven variables considered as significant. However, the explanatory power of these variables on the variance of the communities is very low (global adjusted *R*^2^ = 0.116). In decreasing order of influence, the variables Elevation, CWD, Nitrogen, PH, C/N ratio, FDD, and DRT have respectively an adjusted partial *R*^2^ of 0.026, 0.025, 0.009, 0.007, 0.004, and 0.003.

### Niche Description of the MOTUs Identified at the Species and Genus Levels

**FIGURE 9:**
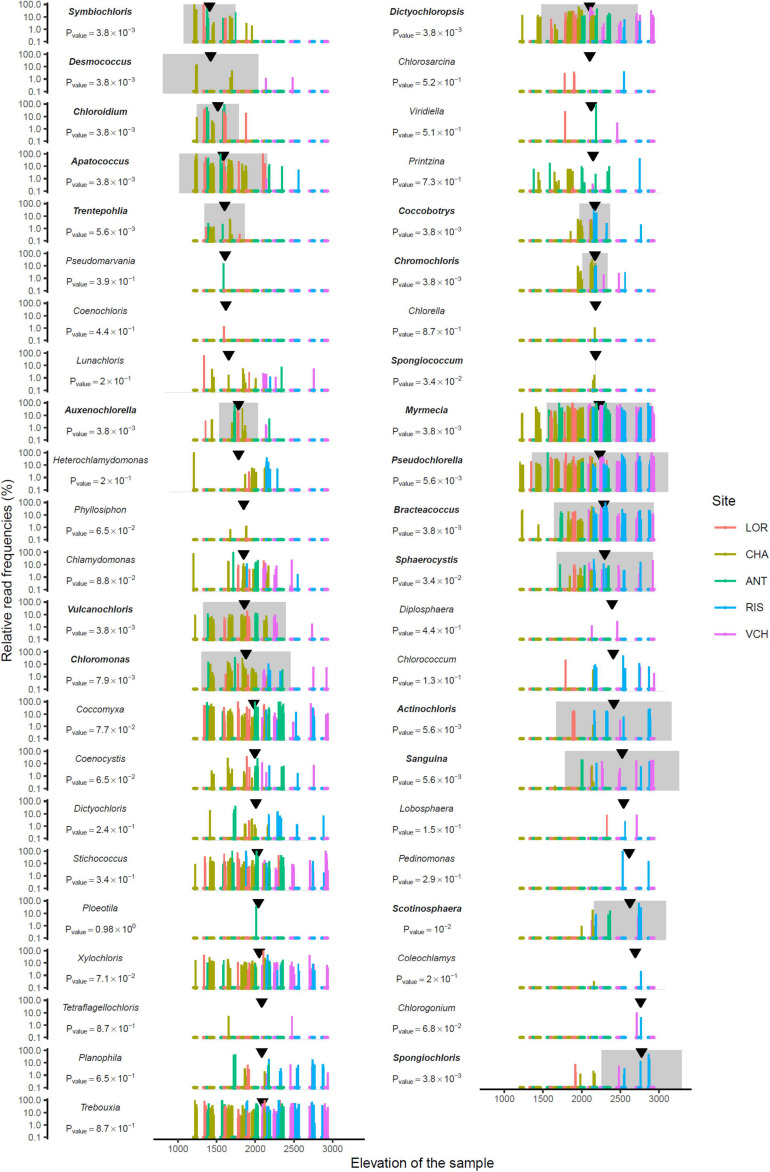
Read relative frequency along elevation for each genus identified. Arrows indicate the median. The range in grey centered on the pic of density is where the MOTU is the most abundant. *P*-values were evaluated using the Mann–Whitney test. Bold taxon names indicate a significant *p*-value at 0.05.

**FIGURE 10:**
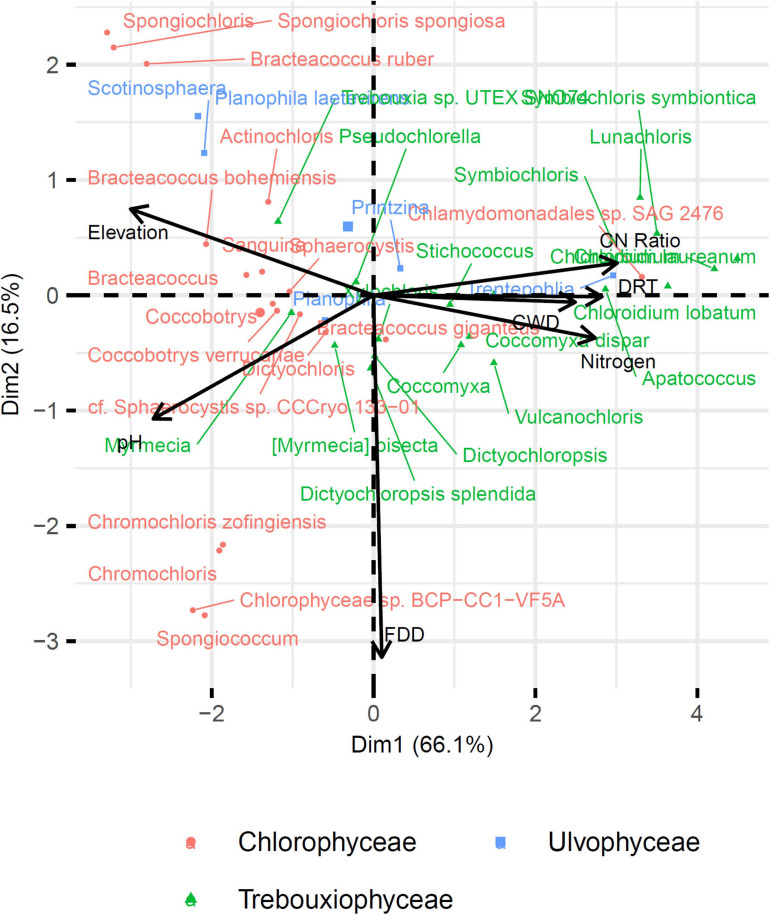
Principal component analysis of the 43 taxa with specialized niche. Taxa are positioned according to the Outlying Mean Index (OMI) of their niche.

Fifty-one and forty-five MOTUs were assigned to a species or genus respectively. For each of these 96 taxa, the optimal range for each of the seven environmental and bioclimatic variables was determined. For each of the variables, it was possible to identify taxa with optimal ranges spanning the entire environmental gradient (Figure 9 and Supplementary Figures 3–15). Figure 9 shows the optimal elevational range of all identified genera, from lowest to highest altitude. While *Symbiochloris*, *Desmococcus*, *Chloroidium*, *Apatococcus*, *Trentepohlia* were associated with low elevation, *Actinochloris*, *Sanguina*, *Scotinosphaera*, and *Spongiochloris* were preferentially found at high elevations (Figure 9). Forty-three of the 96 taxa tested (18 species and 25 genera) had a niche significantly specialized compared to the tested span of environmental variables. The niche of these taxa was compared by performing a Principal Component Analysis (PCA) where each of these taxa was defined by the center of its optimum interval for each of the variables (Figure 10). The two first axes of the PCA carried most of the variance (66.1 and 16.6%, respectively). *Chlorophyceae* were significantly more localized to the left on this axis than *Trebouxiophyceae* (Mann–Whitney *p*-value = 2.5 × 10^–6^). This position corresponded to a preference for higher pH and elevations, whereas *Trebouxiophyceae* prefer a higher C/N ratio and higher nitrogen. This was consistent with the impact of pH and elevation on the relative abundance of these two classes of *Chlorophyta* (Figure 7). The second axis, mainly related to FDD, segregates *Chlorophyceae* taxa, when *Trebouxophyceae* are closer to its center.

## Discussion

This work addressed the potential altitudinal zonation of green algae in mountain areas in temperate regions in the Northern hemisphere, focusing on algae populating soil, either as their natural habitat or as a transient reservoir for dormant cysts, taking the French Alps as study case. Analysis of eDNA allowed the detection of DNA fragments released by broken cells over long periods, mitigating short term temporal variations, and providing access to a “soil memory effect” (Foucher et al., 2020), of interest for such a preliminary study of the spatial distribution of algal communities. To date, and to our knowledge, no such systematic investigation has been attempted. We benefitted from the availability of soil eDNA samples, obtained by the Orchamp consortium. Five gradients have been sampled at distant locations, covering elevations from about 1,250 to 3,000 m (Figure 1). It must be noted that only two of the five gradients reached the niveal zone, nevertheless, they provide information on *Chlorophyta* clades present at the highest elevations. We based our study on several assumptions. The first one is that soil samples could provide information on the presence of species regardless of seasonal variations, based on the above-mentioned “soil memory effect,” which may alter relative abundance, and even determine the absence or presence of some species at the time of sampling. The second assumption was that elevation gradients could be compared, and that an elevation in one site may correspond, approximately, to an elevation in another site.

We hypothesized that, due to their role as primary producers and as pioneer species in open areas, algal DNA could be detected in most sites. The presence of *Chlorophyta* was indeed confirmed in the five elevational gradients, and in most of the soil samples. However, based on a first evaluation using the Euka03 eukaryotic marker, it was clear that *Chlorophyta* DNA occurred in an extremely low proportion (Figure 2 and Table 1). We designed, and validated, two new markers for metabarcoding studies, the *Chlorophyta* phylum marker Chlo01 in the V7 region of the 18S ribosomal RNA, to cover most of the green algae, and the *Chlorophyceae* marker Chlo02 in the 23S ribosomal RNA chloroplast sequence. Both improved our detection of algal DNA and the identification of algal MOTUs, still highlighting an extremely low proportion of *Chlorophyta* DNA in soil samples (Table 1).

The low coverage of green algae might be attributed to the higher proportion of DNA from other organisms, which present a higher biomass. Microbial communities develop in soil away from light exposure, and are therefore expected to be dominated by heterotrophic species feeding off of available organic carbon and other nutrients. Multicellular eukaryotes are also present with substantial levels in biomass, like fungi, animals or plant roots. The low proportion of algal DNA may explain why most of the MOTUs we detected appeared site-specific (Figure 4). This limitation should be taken into account, and hopefully corrected in future, more comprehensive analyses. Here, we therefore considered that the detected clades were probably the most abundant ones in algal communities, and that the distribution patterns we detected reflected strong trends.

Since numerous green algae have the capacity to form airborne spores (Tesson et al., 2016), allowing them to be transported by ascending winds, and since many stressful environmental parameters such as extreme temperatures and high UV light exposure are correlated with elevation, we wondered whether altitudinal zonation may be a major determinant of spatial occupancy and of biodiversity, regardless of the sampling sites. Among the seven non-collinear environmental and bioclimatic variables we monitored, pH, elevation, nitrogen content, FDD, and CWD (Figure 5) were significantly related to diversity. Thus, our prior assumption that elevation could be compared between sites proved to be acceptable, as this parameter appeared as one of the plausible determinants of algal distribution. Nevertheless, it was not sufficient, not even prominent, since pH and nitrogen appeared as likely more important. It must be noted that the CHA gradient showed some atypical results compared to other sites, which may be due to the location of this site, facing a highly dense urban area and likely influenced by winds streaming from the Rhône valley (Figure 1).

When focusing on *Chlorophyta* classes, *Trebouxiophyceae*, and *Chlorophyceae* appeared as the two most abundant ones in all our samples, consistent with their prominence in aero-terrestrial habitats. Their relative abundance was strikingly correlated with soil pH and elevation (Figure 7), highlighting again these two parameters as determinant. We refined our analysis on the 51 and 45 MOTUs we could assign to a species or genus, respectively, attempting to determine the optimal range for each of the four environmental variables.

The distribution at the genus level is not simple to analyze, as genera encompass a number of species, which can be distinct between samples, and/or having overlapping niches hiding more specific distributions at the species levels. Some genera, like *Stichococcus*, *Coccomyxa*, *Xylochlorus*, *Trebouxia*, *Dictiochloropsis*, *Myrmecia*, *Pseudochloroella*, or *Bracteacoccus* were detected at nearly all elevations, and the pattern of their distribution rather suggest that they correspond to cosmopolitan genera (Figure 9). This does not exclude that, within these genera, some species may have emerged as highly specific of certain niches. Further studies, at the species and/or ecotype levels are therefore needed for these large clades. *Desmococcus*, known to comprise species that are tolerant to desiccation (Lüttge and Büdel, 2010) or covering artificial hard surfaces in urban areas in central Europe (Hallmann et al., 2016), are associated with low elevations (Figure 9), possibly connected to a broader geographic distribution in valleys. *Symbiochloris*, comprising free-living and/or lichenized algae (Škaloud et al., 2016) are also associated with low elevation, but data do not allow determining whether corresponding species are lichen photobionts or not. Interestingly, the *Sanguina* genus corresponding to species causing red snow blooms, i.e., *Sanguina nivaloides* and *Sanguina aurantia* (Procházková et al., 2019), is found at elevations higher than ∼2,000 m, with an optimal occurrence at ∼2,400 m. This finding is consistent with the proliferation of *S. nivaloides* and *S. aurantia* in the snow cover encountered at these elevations. It also highlights that the soil can possibly be a long-term reservoir for these snow algae in the summer season. Surprisingly, *Sanguina* distribution did not highlight any significant correlation with the intensity of freezing events, as measured by FDD (Figure 10 and Supplementary Figure 12), which may relate to the specific habitat of this genus, developing in the snow (and underneath soil), at temperatures close to 0°C, regardless of air above, which could reach much lower temperature levels. Eventually, the two genera preferentially found at high elevations were *Scotinosphaera*, described previously in various habitats in low elevations as well (Škaloud et al., 2013) and *Spongiochloris*, based on a small number of occurrence in two sites (Figure 9). The airborne spreading of *Spongiochloris* has been described in previous reports (Tesson et al., 2016), which may explain a transport of this taxon reported in various locations in desertic or mountain sites to such high elevation.

When focusing on species-level MOTUs (Supplementary Figure 3), obtained patterns needs to be considered with caution due to the lack of reference genomes of *Chlorophyta* in existing databases, and possible misannotations. Still, the number of accessions previously recorded in mountain areas or in polar regions is striking, including *Chloromonas nivalis* (optimal elevation at ∼1,800 m; Procházková et al., 2018); *Ploeotila* sp. CCCryo 086-99 (which is closely related to *Sanguina* species; Procházková et al., 2019), detected here in one sample at ∼2,000 m; *Trebouxiophyceae* sp. SC2-2 (first described in glacial refugia in Antarctica; De Wever et al., 2009), here quite cosmopolitan, with an optimal elevation at ∼2,100 m; *Sphaerocystis* sp. CCCryo 133-01 (described in moss fields along snow melt in the Spitzberg, in the CCCryo collection; Leya, 2020), here with an optimal elevation at ∼2,150 m; *Trebouxia* sp. UTEX SNO74 (previously recorded as *Chlamydomonas nivalis*, based on a collection in the snow; Matsuzaki et al., 2018), here with an optimal elevation at ∼2,500 m. In the latter case, the distribution of *Trebouxia* sp. UTEX SNO74 is broad, including occurrence at lower elevation, suggesting that species associated to this accession might be tolerant, but not specific, to the conditions found in high elevations. With optimal elevation higher than 2,500, species include *Planophila laetevirens* (previously detected in various locations in the Alps as well as high latitudes; Schmidt and Darcy, 2015), *Bracteococcus ruber* (recently detected in alpine mountains in New Zealand; Novis and Visnovsky, 2012) and *Spongiochloris spongiosa*. Taxa known to accumulate high levels of carotenoids, such as *Chromochloris zofingiensis* (Ye and Huang, 2020), *Sanguina* (Procházková et al., 2019), or *Bracteacoccus* (Chekanov et al., 2020) species were also found at high altitudes. Interestingly, in one occurrence at ∼3,000 m, *Bracteacoccus aerius* was detected. This species known to stick to dust in air suspension, may have reached the top of this mountain site via ascending winds.

Altogether, obtained data support that species-level MOTUs are likely associated with an altitudinal zonation. Other environmental factors may be also important, in combination, as shown by the distribution patterns we also obtained with pH, nitrogen and C/N (Supplementary Figures 3–9). We do not exclude that the taxonomic assessments presented in this study may be biased, first by a high level of similarity between the amplified DNA with that of a close but different species/accession in the reference database, and secondly by an overrepresentation of psychrophile species in the reference database.

Our search for significant correlations highlighted that clades belonging to the *Chlorophyceae* were distinct from *Trebouxiophycea* by their preference for higher pH and elevations. By contrast, *Trebouxiophyceae* appeared to prefer a higher C/N ratio and higher nitrogen (Figure 10), suggesting that soil nutrients were determinant as well. When we considered all sites, the algal diversity was actually significantly higher in the litter (soil not totally decomposed) compared to the deep soil layer underneath. The litter is richer in nitrogen, carbon and organic matter and is only partially exposed to light. Based on their compositions, soils may therefore favor species being both phototroph and heterotroph, which has been known for a long time to comprise numerous *Chlorophyta* species (Parker et al., 1961). The capacity to combine phototrophy and heterotrophy, and in the case of synergies between these two energetic metabolisms, mixotrophy, seems therefore a possible strategy for algae to spread in the soil, compared to algae from lakes and rivers, which may simply rely on strict phototrophy. This metabolic capacity may therefore also be determinant at the level of genera and/or species.

## Concluding Remarks

Metabarcoding is a tool of choice to study algae communities in such large areas and territories as the different mountain massifs that make up the French Alps. The main difficulty compared to other microbial phyla lies in the lack of molecular markers and the lack of reference genomes in databases. The Chlo01 and Chlo02 green algae markers developed here successfully amplified green algae from Alpine soil samples. They also amplified DNA from marine green algae strains from the RCC public collection used as positive controls. They will be extremely useful for future studies.

The amount of microalgae DNA is very small in the soil. To get a solid overview of the biodiversity of microalgae in the Alpine soil, the sampling effort should be increased as well as the number of PCR technical replicates, resembling the type of effort used for ancient or freshwater DNA (Valentini et al., 2016). Despite this technical limit, we assumed that detected DNA corresponded to the most abundant species, and we were therefore still able to draw some conclusions from this preliminary work. Firstly, our sampling sites allowed us to test whether elevation was a major, if not the most prominent, determinant of spatial distribution, based on the assumption that algae would mainly spread via airborne spores (Tesson et al., 2016) and sit in their preferred habitats under the pressure of parameters correlated with elevation, such as decreasing temperature levels and exposure to increasing UV light. A putative decline of biodiversity due to the extreme conditions in highest elevations was not evidenced. This indicates that photosynthetic eukaryotic algae are present in all niches, and that their diversity can be a source of pioneering species colonizing open areas, such as those opened by the retreat of glaciers. Comparison of read frequency along elevational gradients suggested that elevation, but also pH and soil N in combination contribute to the spatial distribution of green algae. This may be related to the role of pH in the bioavailability of soluble nitrogen (Lee et al., 2006), but one cannot exclude that some species may have optimal pH *preferenda*. Future works will therefore be needed to investigate the impact of the geological context, since all sites investigated here were crystalline and acidic. Different distribution patterns might therefore be encountered in soils covering calcareous and alkaline rocks in pre-alpine massifs. More refine analyses regarding inorganic nitrogen (nitrate, nitrite, and ammonium) are also requested. Vertical differences in green algae biodiversity supported the fact that factors other than light were determinant in the presence of species in soil, possibly acting as essential local reservoirs for a long-term occupancy of this habitat. In particular, the C/N ratios seems determinant in the case of *Trebouxiophyceae*, and future work will be needed to refine the role of this parameter in relation with the energetic metabolism of species, being not only phototrophic, but also heterotrophic and/or mixotrophic. Eventually, since atmospheric CO_2_ proved to influence microbial communities in a pH-dependent manner (Gulliver et al., 2016; Yu and Chen, 2019), a relation with CO_2_ solubility, bioavailability and the presence of carbon concentration mechanisms (CCM) in algal plastids need to be investigated.

At the species/accession level, an altitudinal zonation was evidenced, again with pH being determinant in the distribution pattern in a more refined manner. Some species seem cosmopolitan whereas others appear specific to some elevations and corresponding habitats; it is possible that there is an altitudinal zonation of microbial communities in a broader sense, and that there is a relationship with multicellular organisms who are also specific to certain elevations. Based on this work, some of the accessions we highlighted need to be assigned taxonomically with greater precision, to be considered as potential markers of ecosystems’ evolution. *Sanguina* distribution has also attracted our attention, as it was consistently correlated with elevation, with an occurrence at altitudes higher than 2,000 m a.s.l. It is noteworthy to mention that *Sanguina* distribution pattern was not significantly correlated with the intensity of freezing events (FDD), likely related to its snow habitat which temperature is more constant, at 0°C, protected from strong temperature variations of the air above. In this matter, other taxa appear more correlated with the intensity of freezing events, possibly reflecting different adaptation strategies. Altogether, this study will help drawing up guidelines for future, more robust and precise analyses of environmental green algal DNA, from the analysis of more local patterns in some habitats such as forests, meadows, lakes, streams, glaciers, etc., to larger scale comparisons of remote sites in Alpine massifs. In addition to organic carbon, that seems essential for heterotrophic/mixotrophic species over obligate photoautotrophs, light, N, or pH, other factors like temperature, other nutrients including iron, phosphorus, etc., or the availability of water streaming from the network of rivers, lakes and/or runoff from snow/ice melting, etc., need to be considered as well. Future analyses of this group of primary producers, integrating various spatial and temporal scales could therefore help addressing the evolution of mountain habitats and ecosystems, strongly affected by the effects of climate change.

## Data Availability Statement

The original contributions presented in the study are included in the article/Supplementary Material. The data presented in the study and the complete details of the analysis are available on the GitHub webpage (https://alpalga.github.io/Zonation/). All the scripts used for the data analysis and the production of every figure are available on GitHub in the Alpalga/Zonation repository (https://github.com/Alpalga/Zonation).

## Author Contributions

AM-S and WT collected the samples along the Orchamp gradients. LG, AM-S, and DR performed DNA extractions and dilutions. AD-S and DR performed PCRs. FB, AD-S, and EC performed data filtering. AD-S and EC performed data analyses. FP provided expertise in environmental DNA analyses. J-GV, EM, and EC conceived the project. AD-S, EM, and EC contributed to the writing of the manuscript. All authors contributed to the article and approved the submitted version.

## Conflict of Interest

The authors declare that the research was conducted in the absence of any commercial or financial relationships that could be construed as a potential conflict of interest.

## Footnotes

1 https://orchamp.osug.fr/home

2 https://www.ncbi.nlm.nih.gov

3 http://metabarcoding.org/ecopcr

4 https://git.metabarcoding.org/obitools/ROBIBarcodes

5 http://biotools.nubic.northwestern.edu/OligoCalc.html

6 http://metabarcoding.org/ecoprimers

7 http://www.roscoff-culture-collection.org

8 https://www.mn-net.com/

9 https://www.qiagen.com/

10 https://git.metabarcoding.org/obitools/ROBITools
