# Supplementary material for: Altitudinal Zonation of Green Algae Biodiversity in the French Alps
Source: Front Plant Sci. 2021 Jun 7;12:679428. doi: 10.3389/fpls.2021.679428 (PMC8215661; doi:10.3389/fpls.2021.679428)
Supplement: Supplementary file 1 [file Data_Sheet_1.PDF]

Altitudinal zonation of green algae biodiversity in the French Alps

Adeline Stewart, Delphine Rioux, Fréderic Boyer, Ludovic Gielly, François Pompanon, Amélie Saillard, Wilfried Thuiller, The ORCHAMP Consortium, Jean-Gabriel Valay, Eric Maréchal, Eric Coissac

Supplementary Material

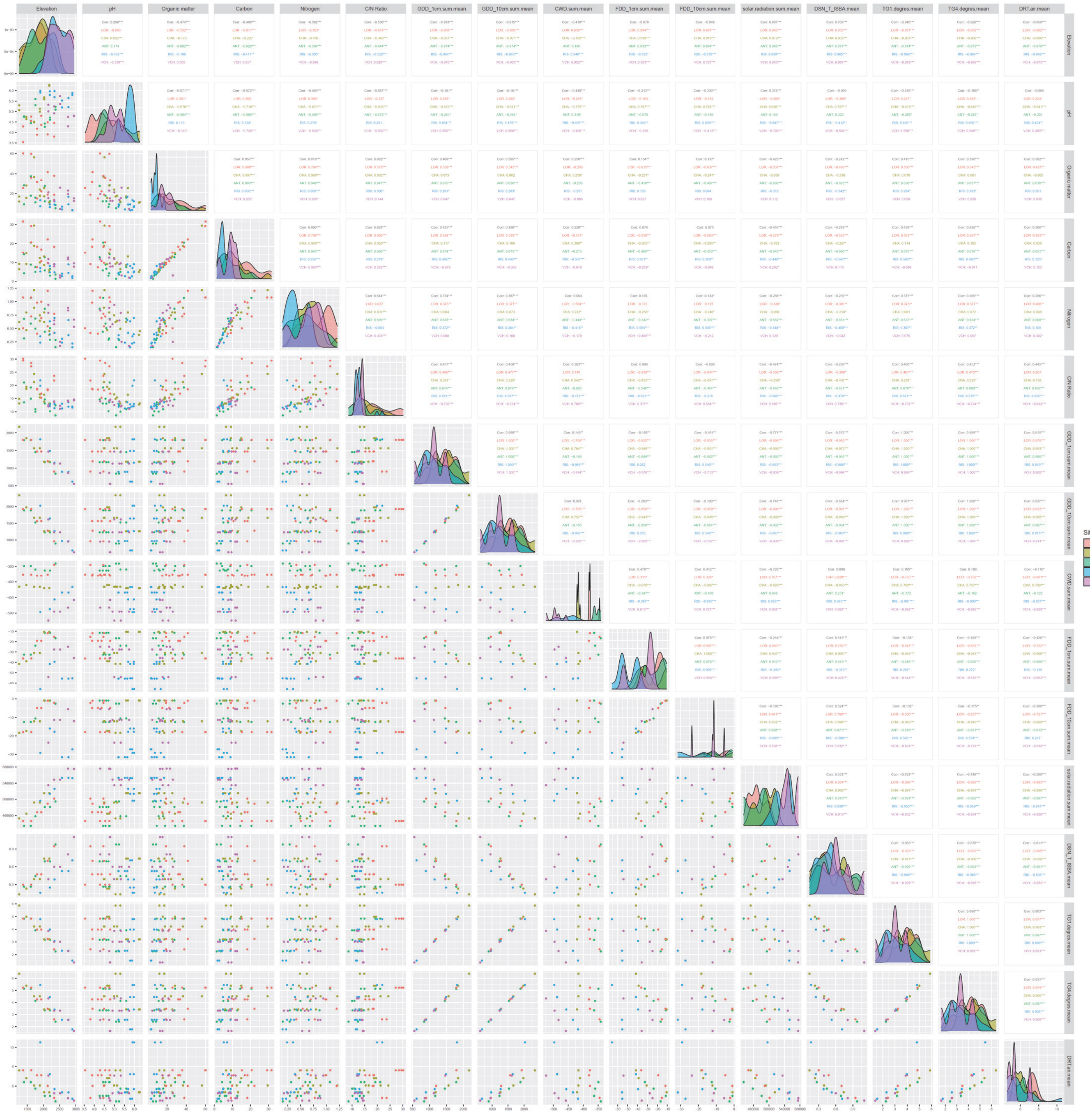

Figure S1. Distributions of environmental variables and their relationships. The density diagrams on the diagonal show the distribution of each variable across the different sampling sites. The scatter plots in the lower triangle of the matrix show the relationships between the variables, and the panels in the upper triangle summarize the linear correlations between the variables. The colors are related to the sampling site and the correlation panels provide their relationship to the sampling sites.

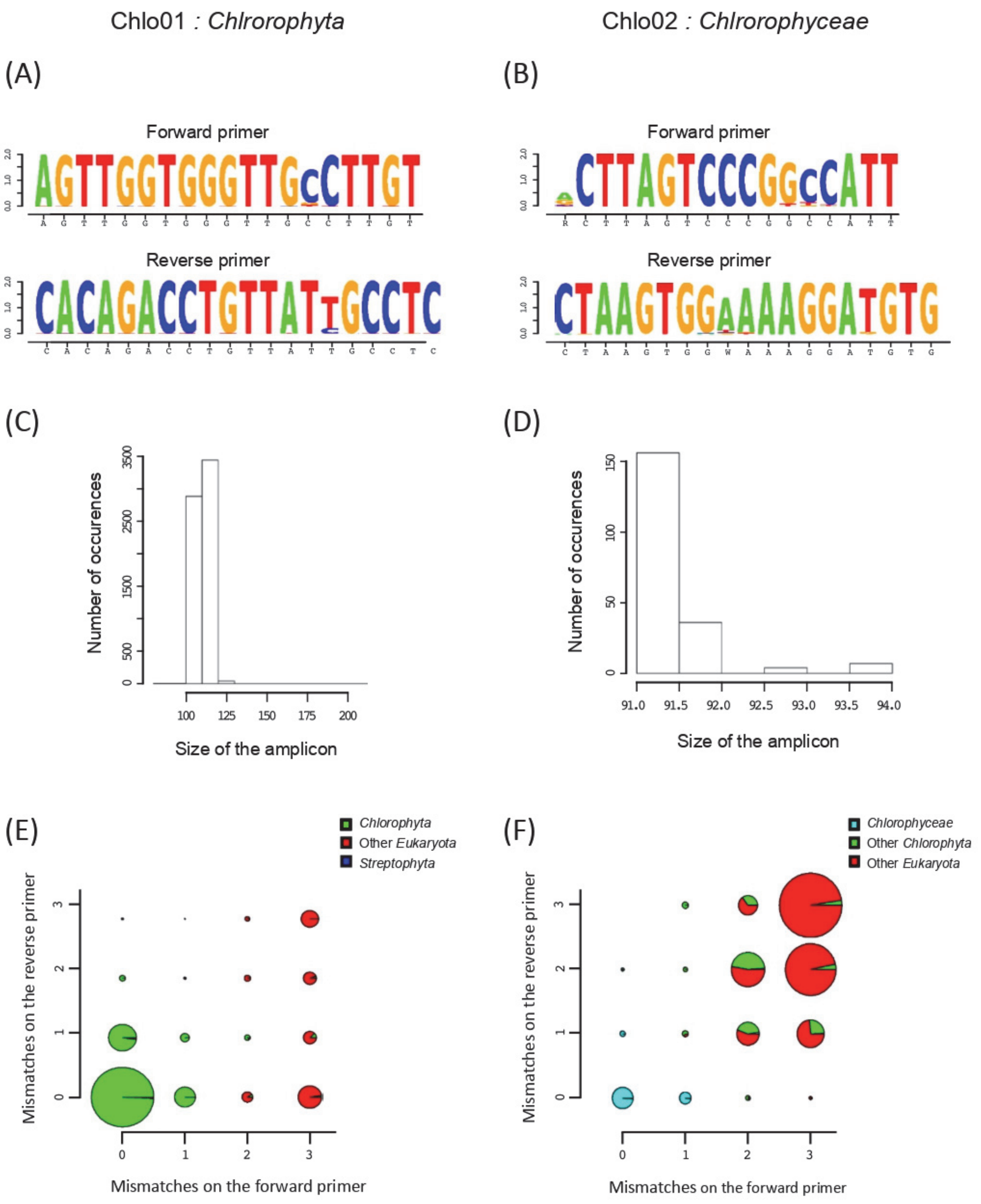

Figure S2. Markers for *Chlorophyta* (Chlo01) and *Chlorophyceae* (Chlo02) for metabarcoding. A, C, E: *Chlorophyta*. B, D, F: Chlorophyceae marker. A, B: Primer sequences. The size of the letters represents the degree of conservation across tested algae sequences. C, D: amplicon size and their respective occurrence across tested algae sequences. E, F: Proportion of target vs non-target taxa amplified depending on the number of mismatches allowed on primer sequences.

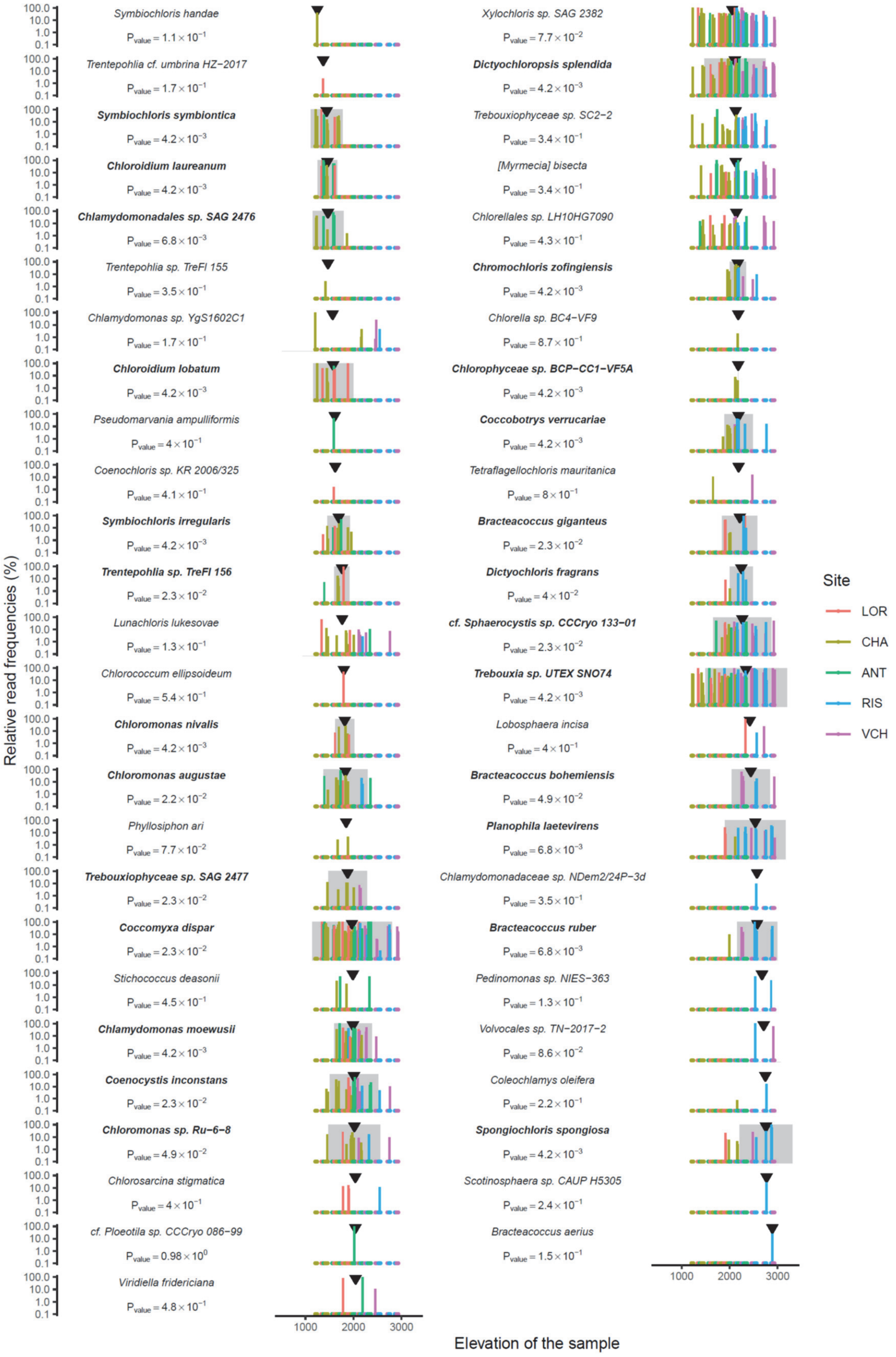

Figure S3. Distribution of species along elevation gradient.

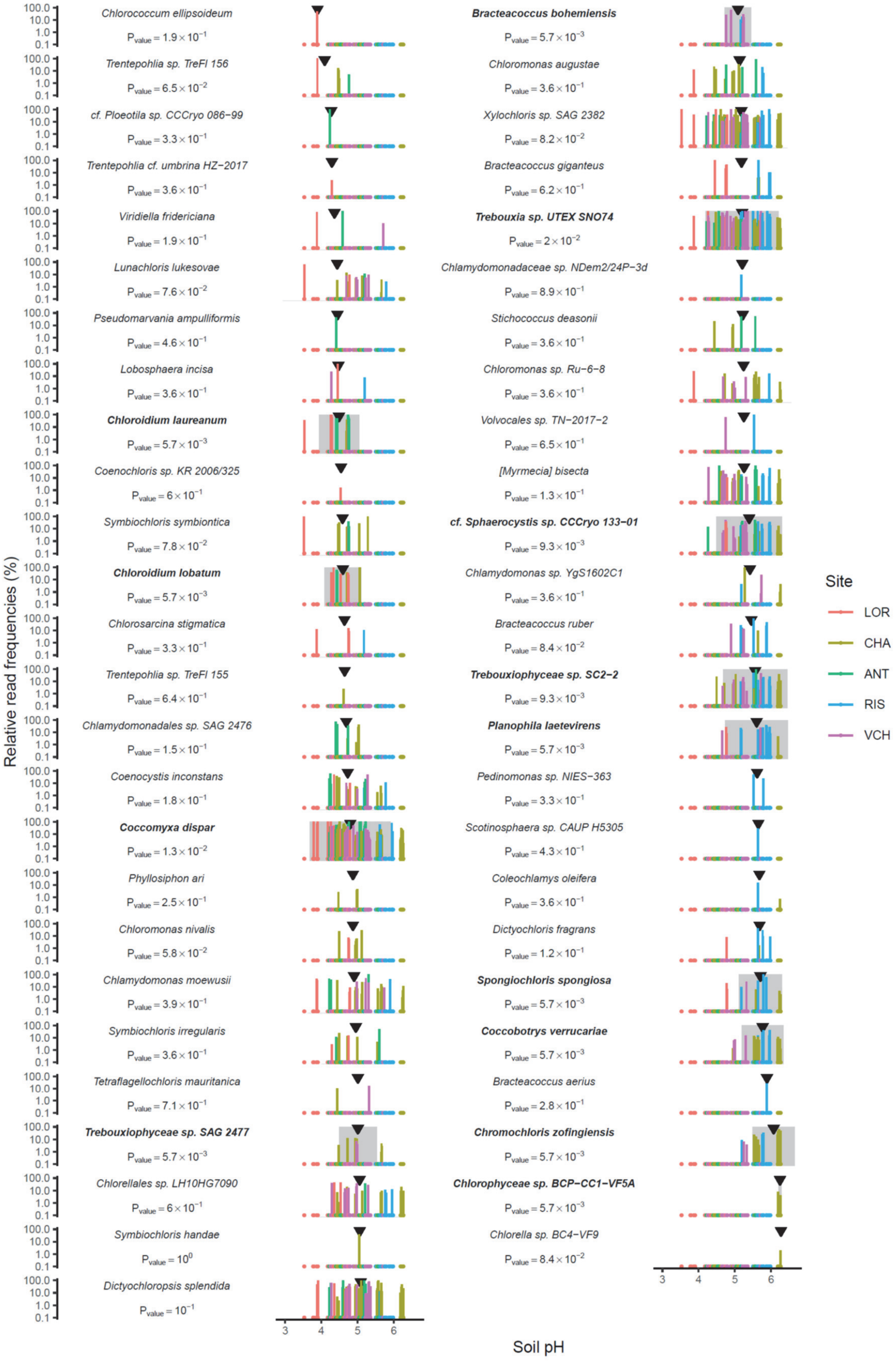

Figure S4. Distribution of species along pH gradient.

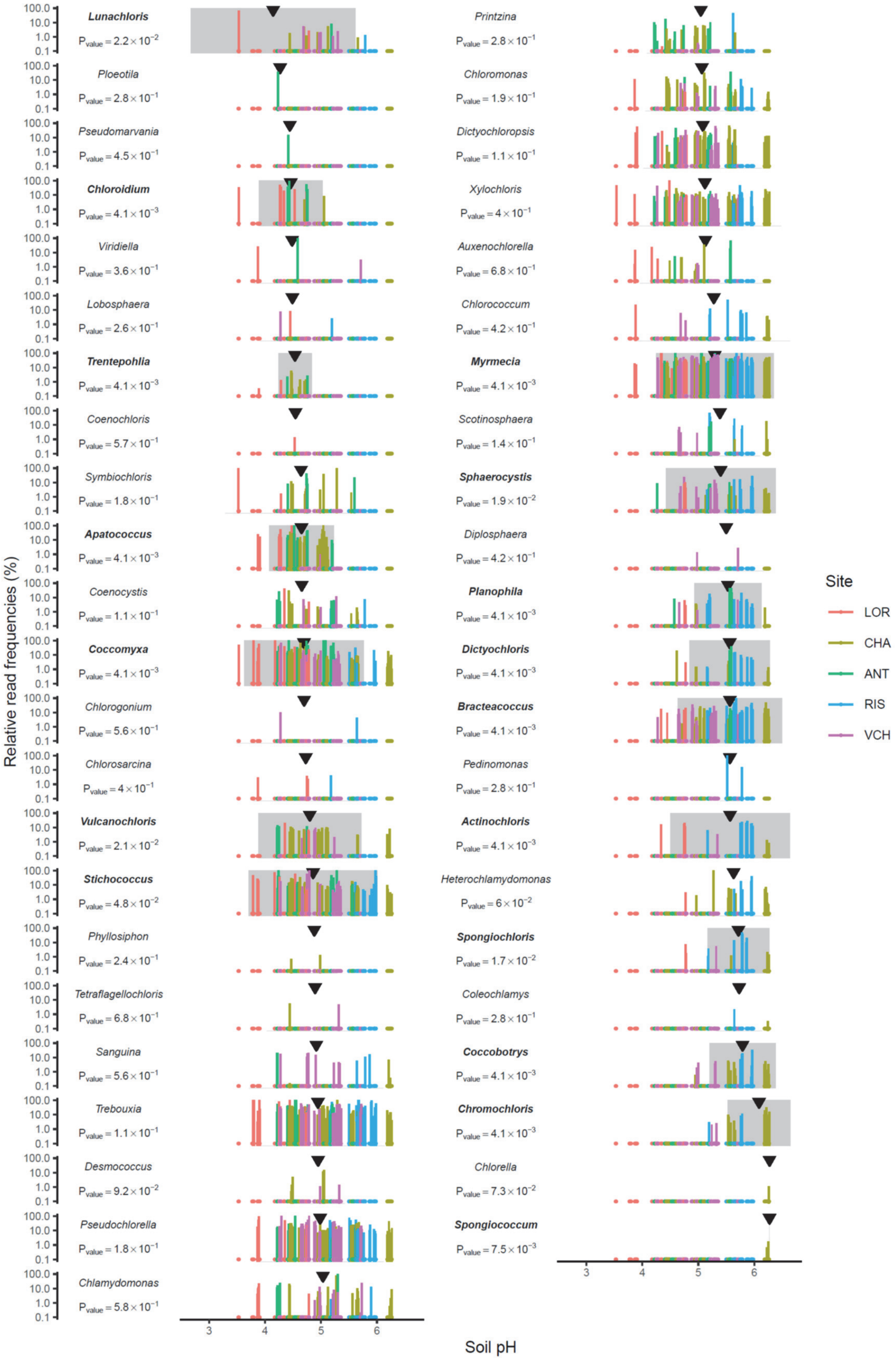

Figure S5. Distribution of genera along pH gradient.

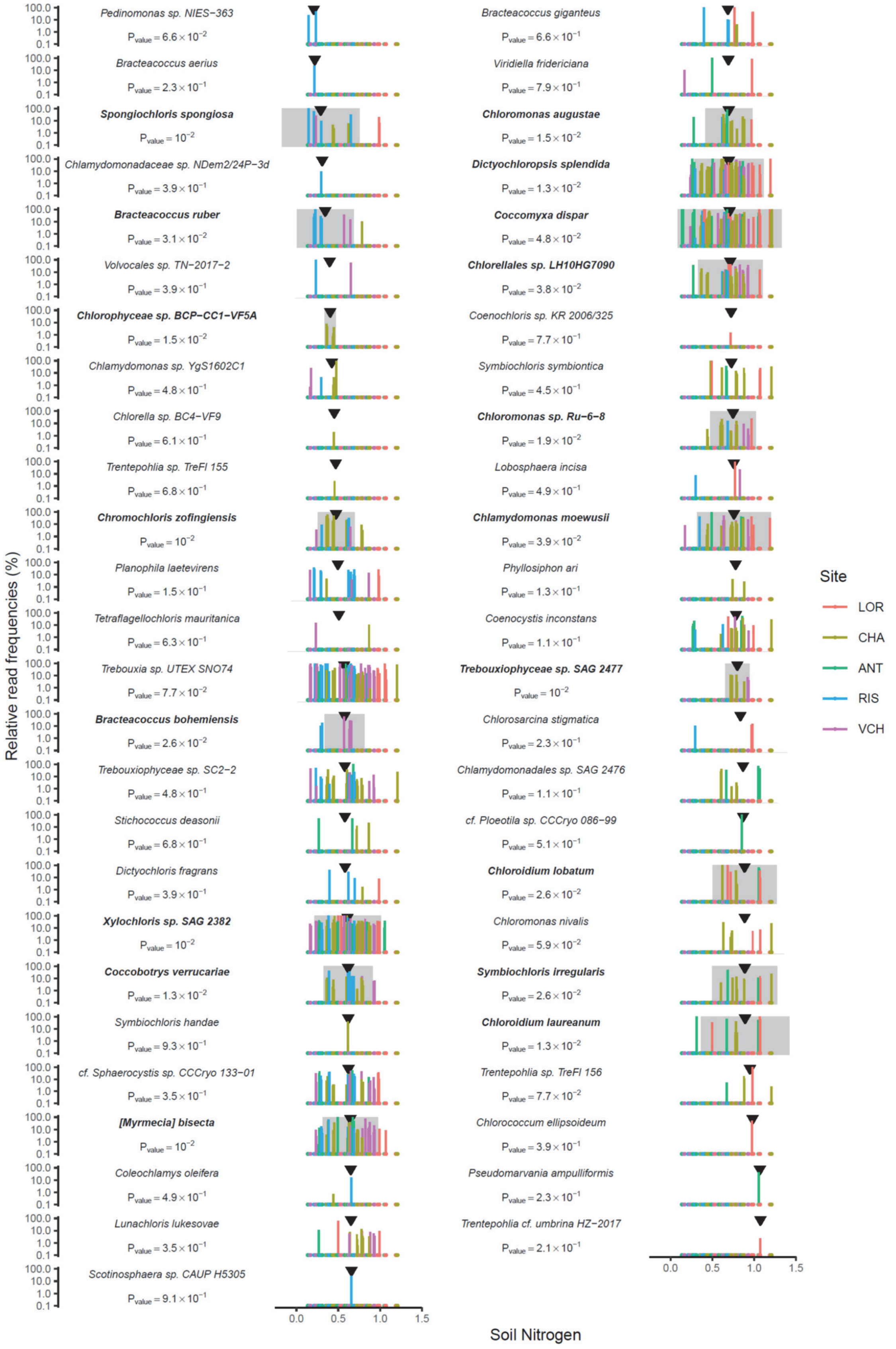

Figure S6. Distribution of species along Nitrogen gradient.

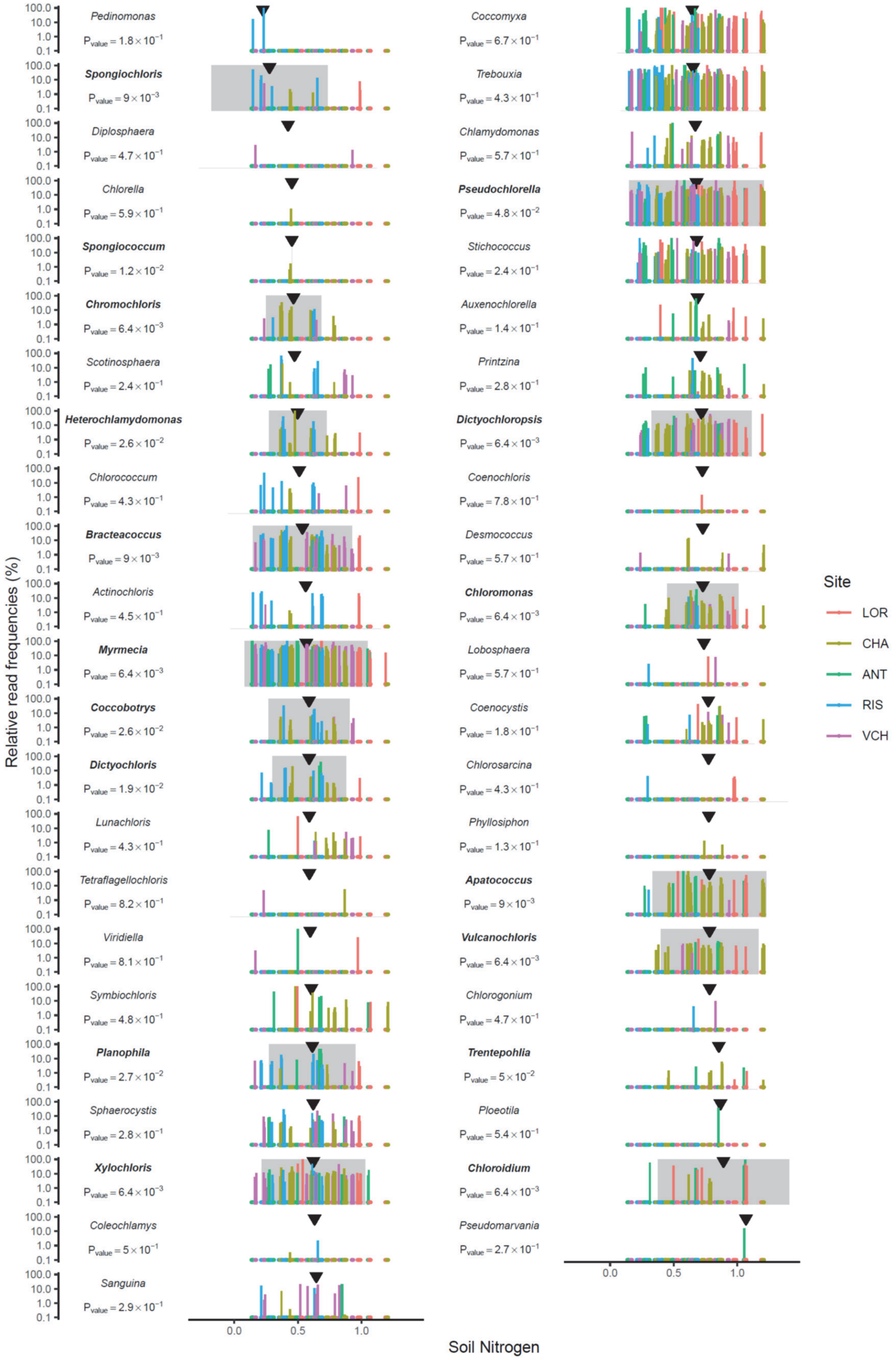

Figure S7. Distribution of genera along Nitrogen gradient.

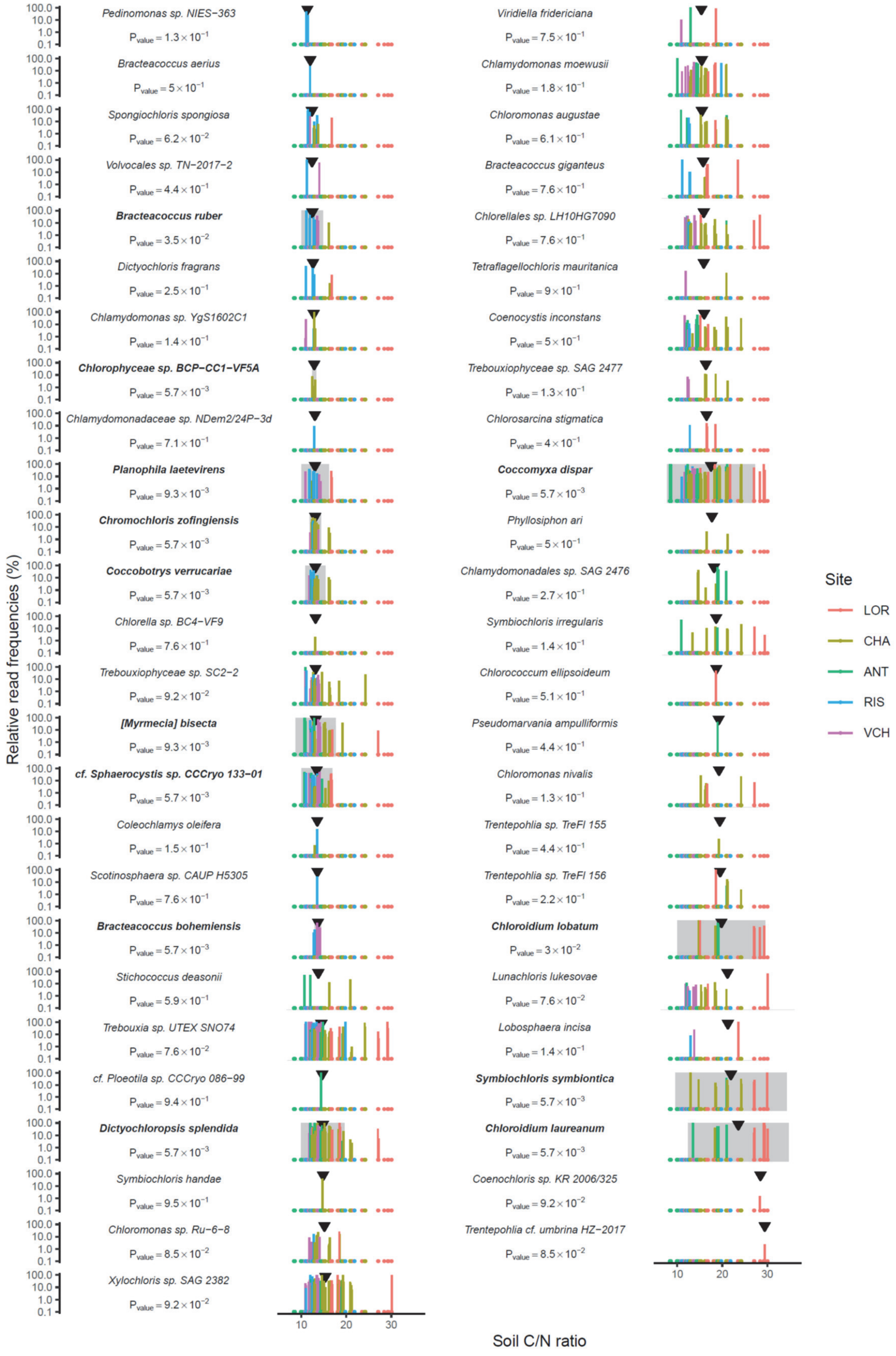

Figure S8. Distribution of species along C/N ratio.

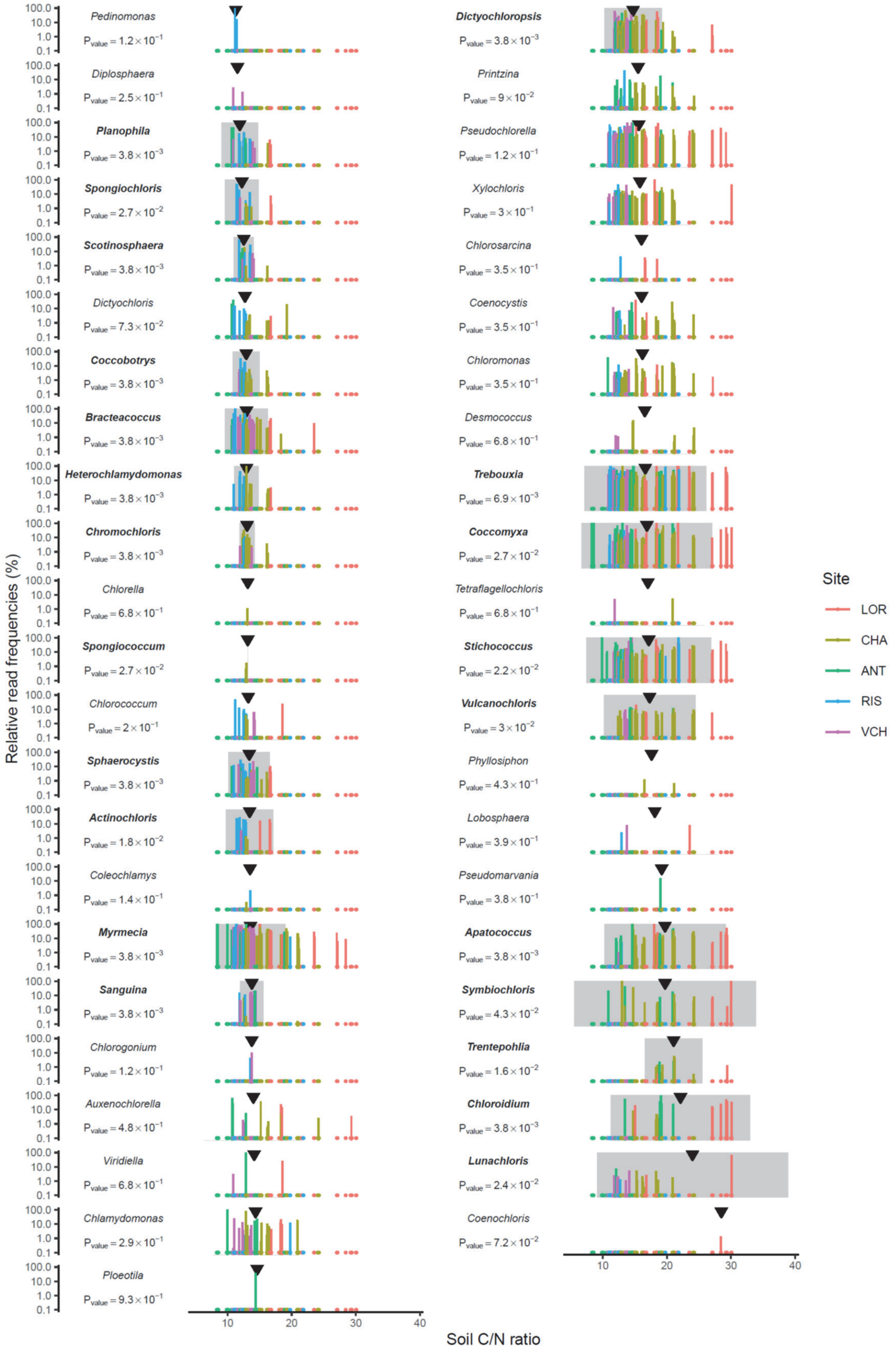

Figure S9. Distribution of genera along C/N ratio.

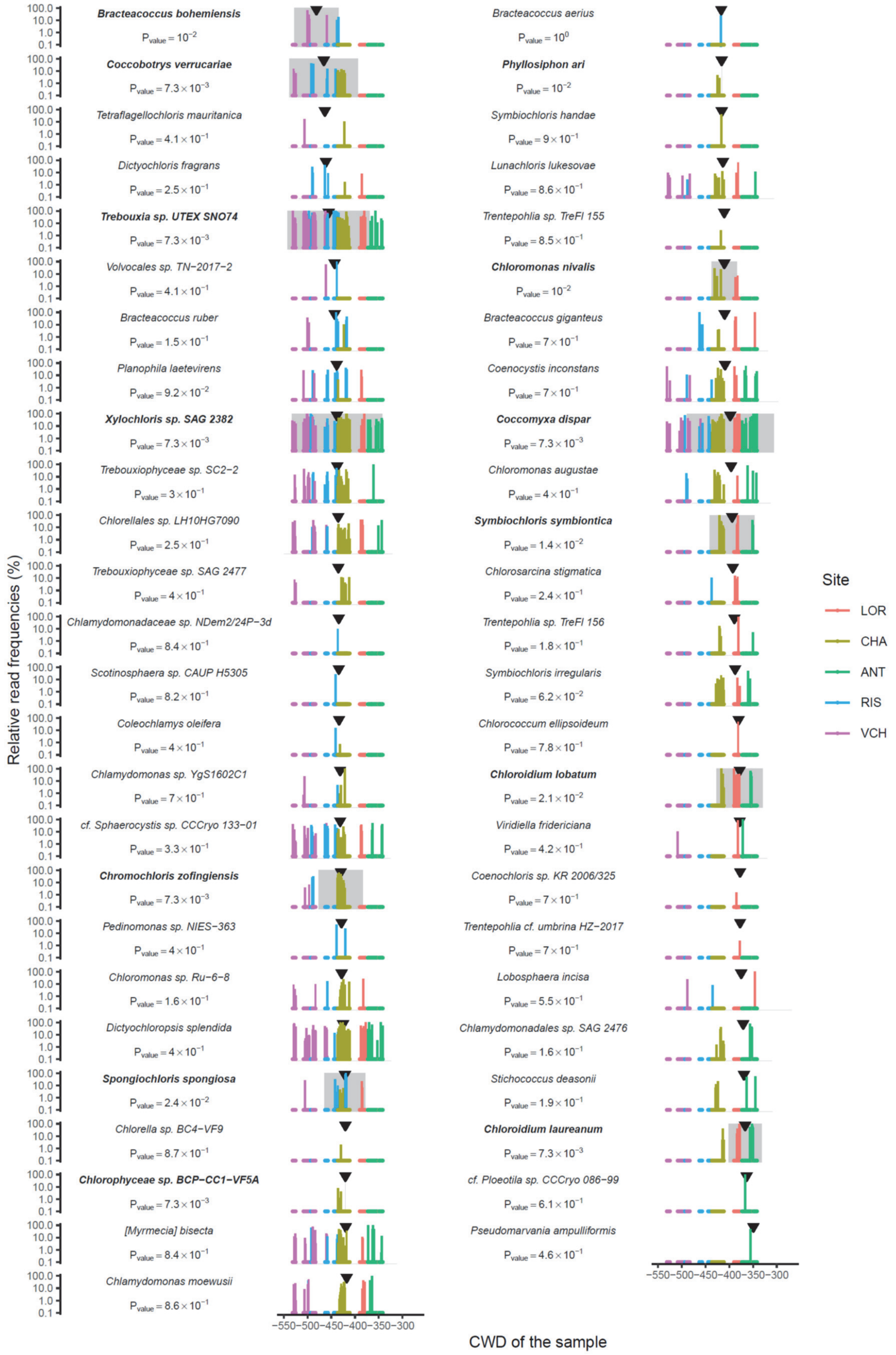

Figure S10. Distribution of species along CWD gradient.

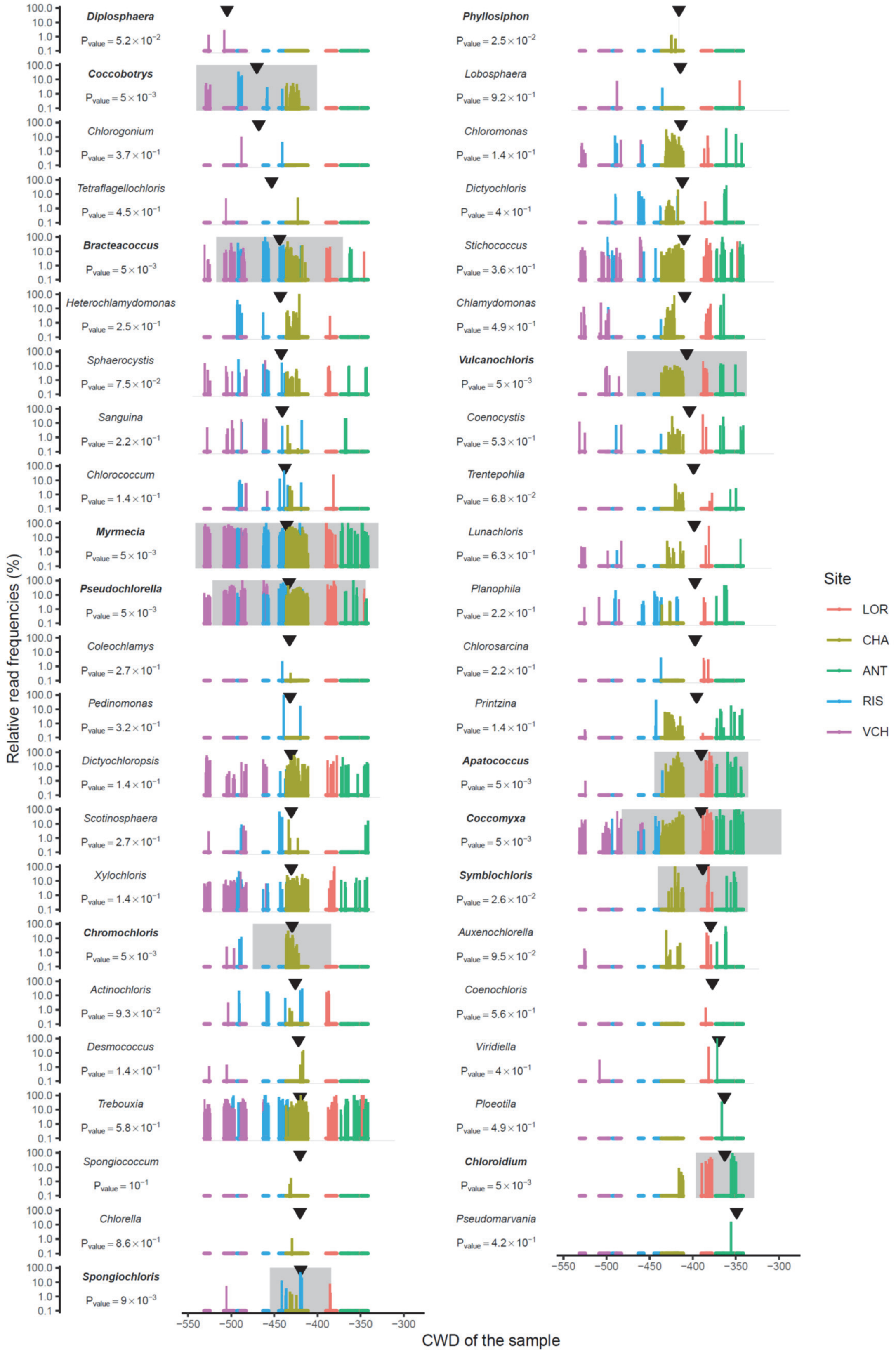

Figure S11. Distribution of genera along CWD gradient.

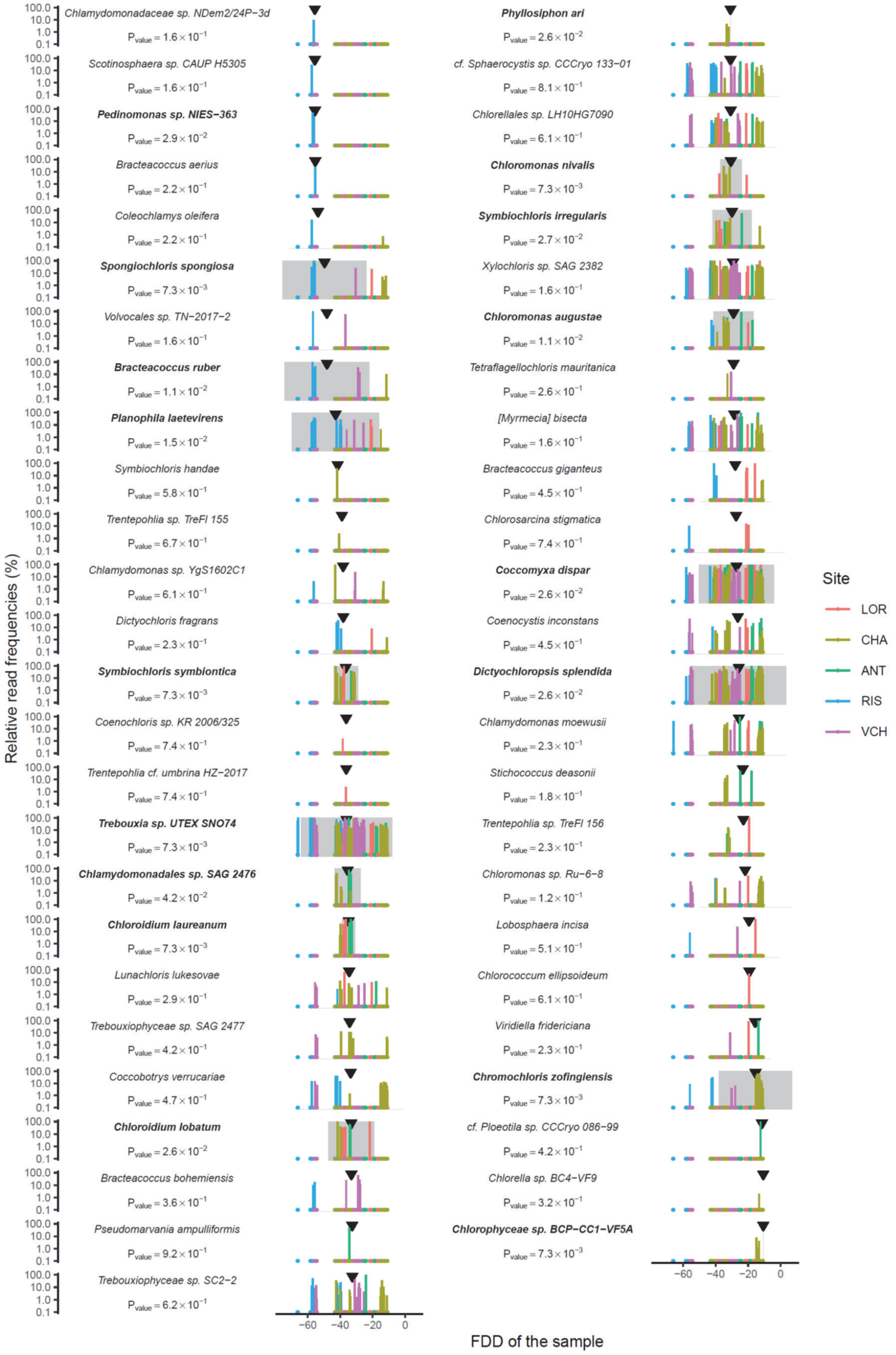

Figure S12. Distribution of species along FDD gradient.

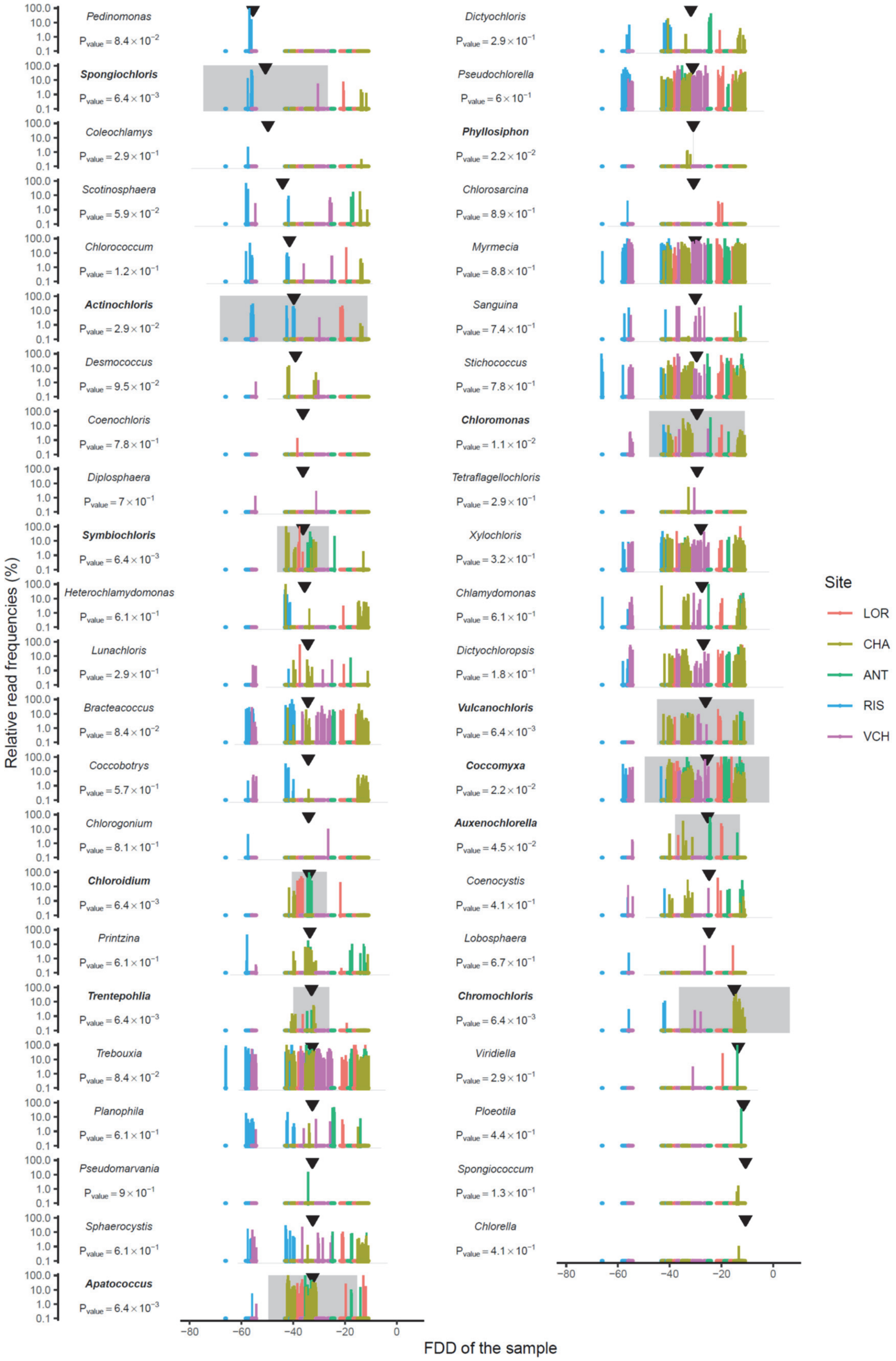

Figure S13. Distribution of genera along FDD gradient.

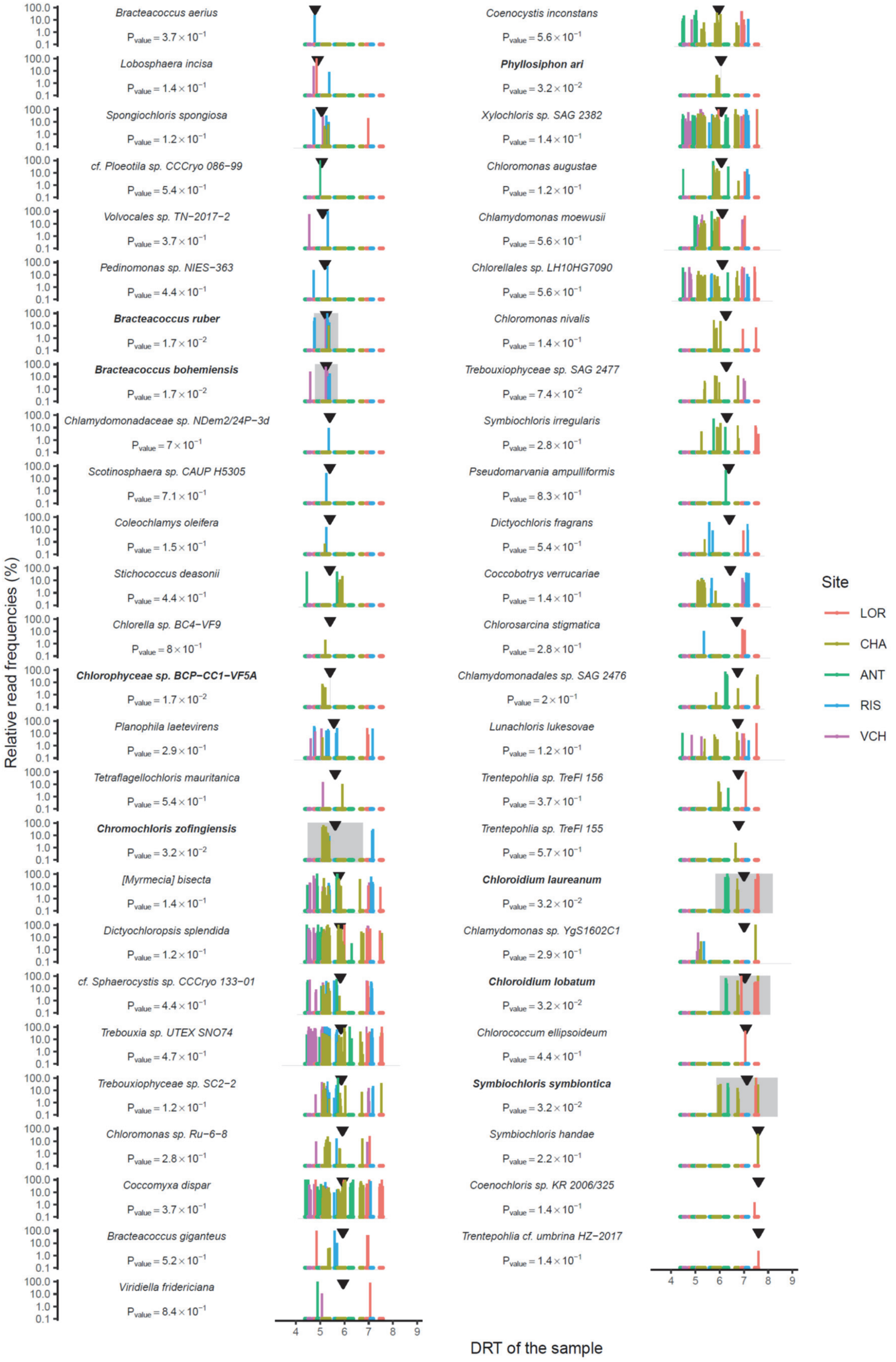

Figure S14. Distribution of species along DRT gradient.

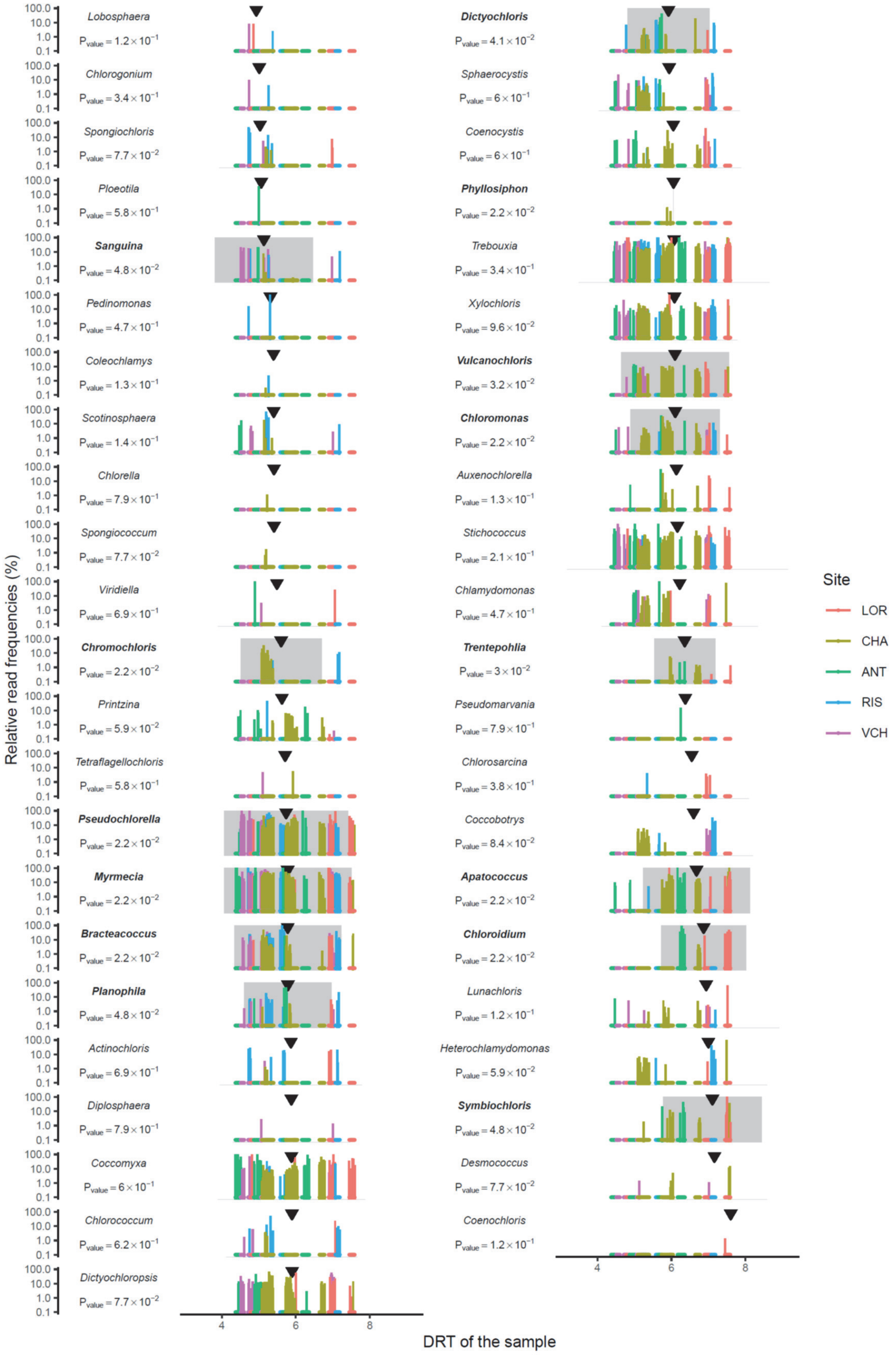

Figure S15. Distribution of genera along DRT gradient.

Table S1. Roscoff Culture collection (RCC) green microalgae selected as controls for the present study; Concentrations and quantity of DNA added to the Positive control Mix in PCRs for metabarcoding are indicated below resulting in a two-fold serial dilution.

RCC ID	Taxid	Taxon	Euka03	Chlo01	Chlo02	DNA quantity (ng)
RCC 7	133490	*Picochlorum atomus*	+	+	-	8000
RCC 443	3052	*Chlamydomonas sp*	+	+	+	4000
RCC 130	3165	*Tetraselmis striata*	+	+	-	2000
RCC 6	3047	*Dunaliella tertiolecta*	+	+	+	1000
RCC 537	114055	*Chlorella vulgaris*	+	+	-	500.0
RCC 581	41880	*Pycnococcus provasoli*	+	+	-	250.0
RCC 891	41891	*Coccomyxa sp*	+	+	+	125.0
RCC 1055	29646	*Stichococcus sp*	+	+	-	62.50
RCC 1563	34154	*Tetraselmis convolutae*	+	+	-	31.25
RCC 2501	36882	*Pyramimonas sp*	+	+	-	15.63
RCC 2960	188557	*Acrochaete sp*	+	+	-	7.813
RCC 3402	88271	*Picocystis salinarum*	+	+	-	3.906
RCC 4743	1418015	*Pseudochloris* sp	+	+	-	1.953
